# Marine Antimicrobial Peptides: From Ocean Biodiversity to Genome Mining, Multi-Omics Discovery, and Biotechnological Innovation in the Battle Against Antimicrobial Resistance

**DOI:** 10.1007/s12602-026-10959-2

**Published:** 2026-04-07

**Authors:** Hassan Aboul-Ella, Heba Aboul-Ella, Mahanama De Zoysa

**Affiliations:** 1 Department of Microbiology, Faculty of Veterinary Medicine, Cairo University, Giza, 12211 Egypt; 2 Department of Pharmacognosy, Faculty of Pharmacy and Drug Technology, Egyptian Chinese University (ECU), Cairo, Egypt; 3 College of Veterinary Medicine and Research Institute of Veterinary Medicine, Chungnam National University, Yuseong-Gu, Daejeon, 34134 Republic of Korea

**Keywords:** Marine AMPs, Peptide engineering, Recombinant expression, Nanodelivery, Genome mining, Aquaculture

## Abstract

Antimicrobial resistance (AMR) has become a significant global health crisis, endangering human health and economic stability. Because traditional antibiotics are no longer effective against pathogens that are resistant to multiple drugs, new antimicrobial strategies are urgently needed. Marine ecosystems, which include the most diverse and extreme habitats on Earth, are home to a huge number of antimicrobial peptides (AMPs) that have unique structures and functions. Many marine AMPs have unique qualities that set them apart from land-based AMPs. For example, they can tolerate high levels of salt, modulate the immune system, and work against a wide range of pathogens. This review gives a broad look at marine-derived AMPs, covering their variety, types, how they work, and how they resist. Biotechnological advances, including omics-guided discovery, recombinant expression systems, peptide engineering, and nanodelivery platforms, are changing AMPs from molecules that haven't been studied much into products that can be made in large quantities and improved. The current work also points out that they can be used in biomedicine as new anti-infective and immunomodulatory drugs, in aquaculture as long-lasting alternatives to antibiotics for managing fish health, and in industrial biotechnology as natural preservatives and agents for controlling biofilm. The merging of marine biotechnology, synthetic biology, and computational peptide design looks very promising for solving the problems of stability, cost, and delivery that we have now. Marine AMPs are likely to be important parts of the next generation of antimicrobial strategies against AMR because they combine marine biodiversity with modern engineering tools.

## Introduction

Bacterial AMR, driven by multidrug-resistant (MDR) and extensively drug-resistant (XDR) pathogens, has emerged as one of the greatest global health threats of the twenty-first century. Once confined largely to hospital environments, resistant pathogens are now widespread in the community, creating a critical public health concern. The rapid spread of AMR is fueled by globalization, the excessive and uncontrolled use of antibiotics in human medicine, animal husbandry, and aquaculture, and the overreliance on broad-spectrum drugs combined with weak antimicrobial stewardship. As a result, treatment options are dwindling, and it is projected that without novel interventions, effective antibiotics may be exhausted by 2050 [1–6].

The maritime environment, encompassing over 70% of the Earth's surface, constitutes the largest and most diverse ecosystem, yet is significantly underexplored relative to terrestrial areas. Marine species, flourishing under harsh conditions of salt, temperature, pressure, and nutrient scarcity, have developed distinctive metabolic and structural adaptations. The production of AMPs encompasses unique attributes, including salt tolerance [7–9], significant immunomodulatory activity [10, 11], chitin-binding capacity [12, 13], and various post-translational modifications or peptide variants derived from fragmentation that exhibit enhanced bioactivity [14–17].

Marine invertebrates constitute a significant reservoir of AMPs, with increasing evidence from molluscs, crustaceans, annelids, echinoderms, cnidarians, and tunicates. Notable instances encompass mussel myticins/mytilins, defensin-like peptides, crustacean crustins, penaeidins, annelid arenicins, and tunicate styelins/clavanins, all of which exhibit extensive antibacterial, antifungal, and anti-biofilm properties, hence aiding in mucosal and hemolymph defense [18–28], in conjunction with peptides derived from marine invertebrate venoms and secretions [29–40]. These traits collectively distinguish marine AMPs from terrestrial counterparts and underscore their significant biotechnological potential. Recent research underscores the influence of habitat pressure [41, 42], microbiome exposure [43, 44], and pathogen-driven selection [37] on the composition of AMP repertoires, affirming their significance as bioactive scaffolds for biotechnology and therapeutics [45].

While numerous reviews have catalogued the discovery and properties of marine AMPs, this work provides a forward-looking, integrated perspective. We underscore the comprehensive translational pipeline—connecting omics-informed discovery, computational and AI-driven design, recombinant and synthetic production methodologies, and nanotechnology-facilitated delivery systems—and assess them through a time-to-application perspective. Recent preclinical studies illustrate the significant translational potential of marine AMPs, such as piscidin-1, epinecidin-1, and halocyntin, which display in vivo efficacy against multidrug-resistant bacteria and retain structural integrity in high-salinity conditions. Moreover, advancements in AI-driven peptide discovery—specifically deep-learning methodologies for AMP identification and design [46] and molecular de-extinction techniques that reconstruct effective antibiotic candidates from genomic data [47]—are broadening the available chemical landscape and expediting the development of next-generation marine-derived therapeutics.

This cohesive framework organizes the review, progressing from sources and mechanisms to resistance and engineering, and culminating with practical applications. This review focuses exclusively on AMPs derived from marine species. The coverage encompasses their inherent diversity, modes of action, immunological functionality, resistance characteristics, and advancements in translational biotechnology, with a deliberate emphasis on concrete examples from fish, marine invertebrates, marine microorganisms, and algae.

## Methods for Literature Selection

A systematic literature review was conducted in PubMed [48], Scopus [49], and Web of Science Core Collection [50] from December 2024 to June 2025, utilizing combinations of the keywords “marine antimicrobial peptides”, “marine AMPs”, “peptide engineering”, “nanodelivery”, “recombinant expression”, and “antimicrobial resistance”. Relevant papers' reference lists were also examined. The inclusion criteria encompassed peer-reviewed articles, reviews, and primary studies detailing the discovery, characterization, or application of marine-derived AMPs,the exclusion criteria comprised non-English publications, conference abstracts lacking peer review, and studies devoid of mechanistic or application relevance. Evidence was systematically categorized by source organism (microorganisms, invertebrates, fish, and algae, mechanism of action, and biotechnological methodology to ensure comprehensive coverage of fundamental biology and translational perspectives.

## Diversity and Classification of Marine AMPs

Marine ecosystems host an extraordinary diversity of organisms that have evolved AMPs as part of their innate defense strategies [27, 51]. Marine microorganisms—including bacteria, actinomycetes, and fungi—represent a prolific source of novel AMPs with unique structural scaffolds and bioactivities [52]. These peptides often exhibit notable stability and performance under extreme marine conditions (e.g., high salinity or pressure), supporting their appeal for biotechnological applications [27, 51, 53]. Marine invertebrates, such as sponges, tunicates, mollusks, and crustaceans, produce a wide variety of AMPs that protect against microbial colonization in microbe-rich environments [54]. Many of these peptides, including defensin- and tachyplesin-like molecules, have been widely studied for strong antimicrobial and immunomodulatory properties [55–57]. Teleost fish also secrete numerous AMPs, such as piscidins and epinecidins, which are central to mucosal immunity and directly relevant to aquaculture health management [58–60]. Finally, marine algae are emerging as a valuable but underexplored source of bioactive peptides and peptide-rich extracts,algal AMPs and peptides frequently show multifunctional roles—combining antimicrobial action with antioxidant and anti-inflammatory effects—broadening their potential for pharmaceutical and nutraceutical applications [61, 62]. Collectively, these diverse sources highlight the vast and still underexploited reservoir of marine AMPs that can be harnessed for biomedical, aquaculture, and industrial biotechnology [51, 54].

**Fig. 1:**
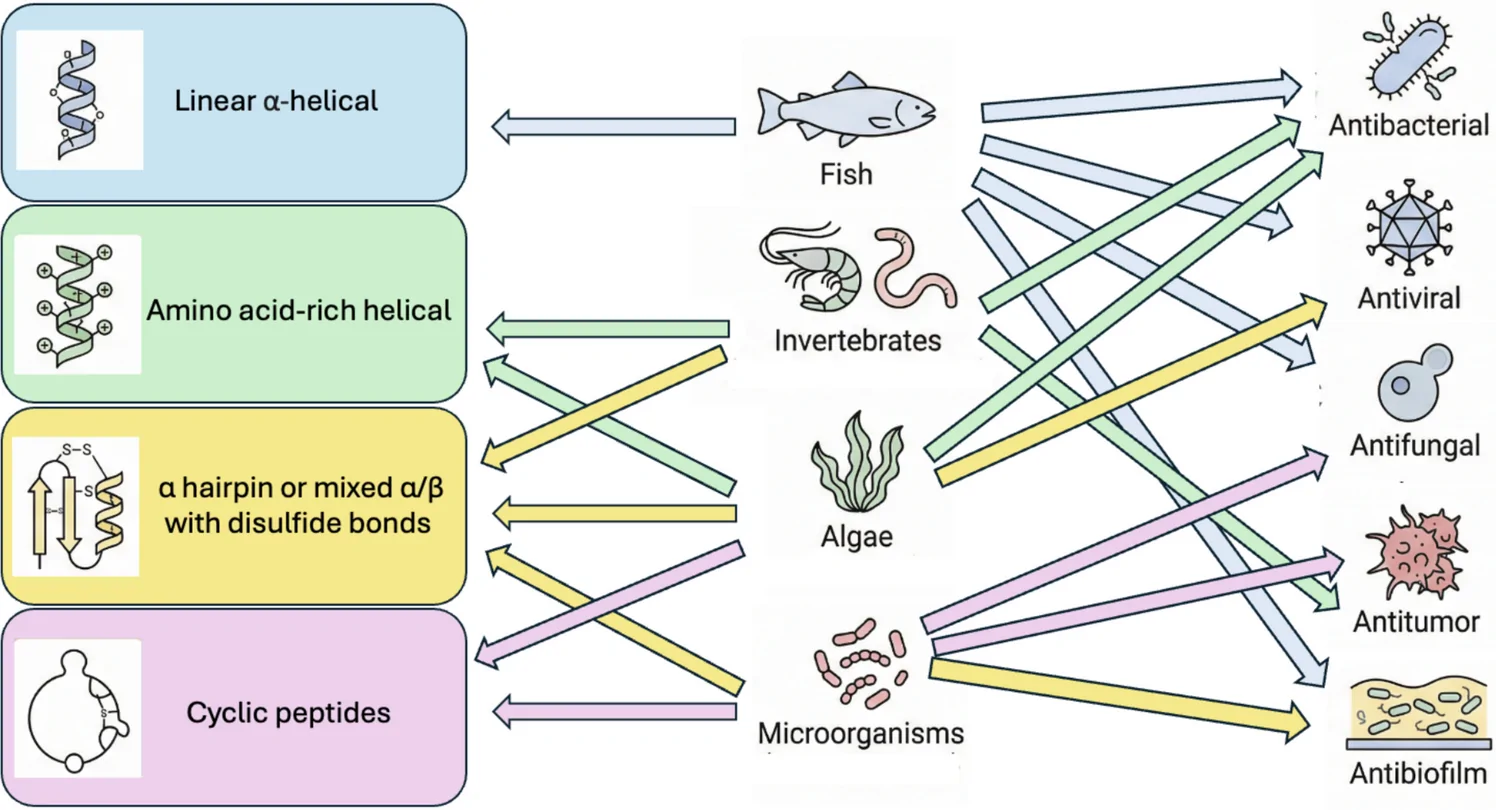
Classification and overview of structural classes, biological sources, and major activities of marine antimicrobial peptides (AMPs). Marine organisms, including fish, invertebrates, algae, and microorganisms, produce AMPs that can be grouped into linear α-helical peptides, amino–acid–rich helical peptides, β-hairpin or mixed α/β peptides stabilized by disulfide bonds, and cyclic peptides. These structural classes are associated with broad antimicrobial and immunomodulatory functions, including antibacterial, antiviral, antifungal, antibiofilm, and antitumor activities, as indicated by the connecting arrows. Key AMP families per group are discussed in the diversity and classification of marine AMPs, with citations

**Fig. 2:**
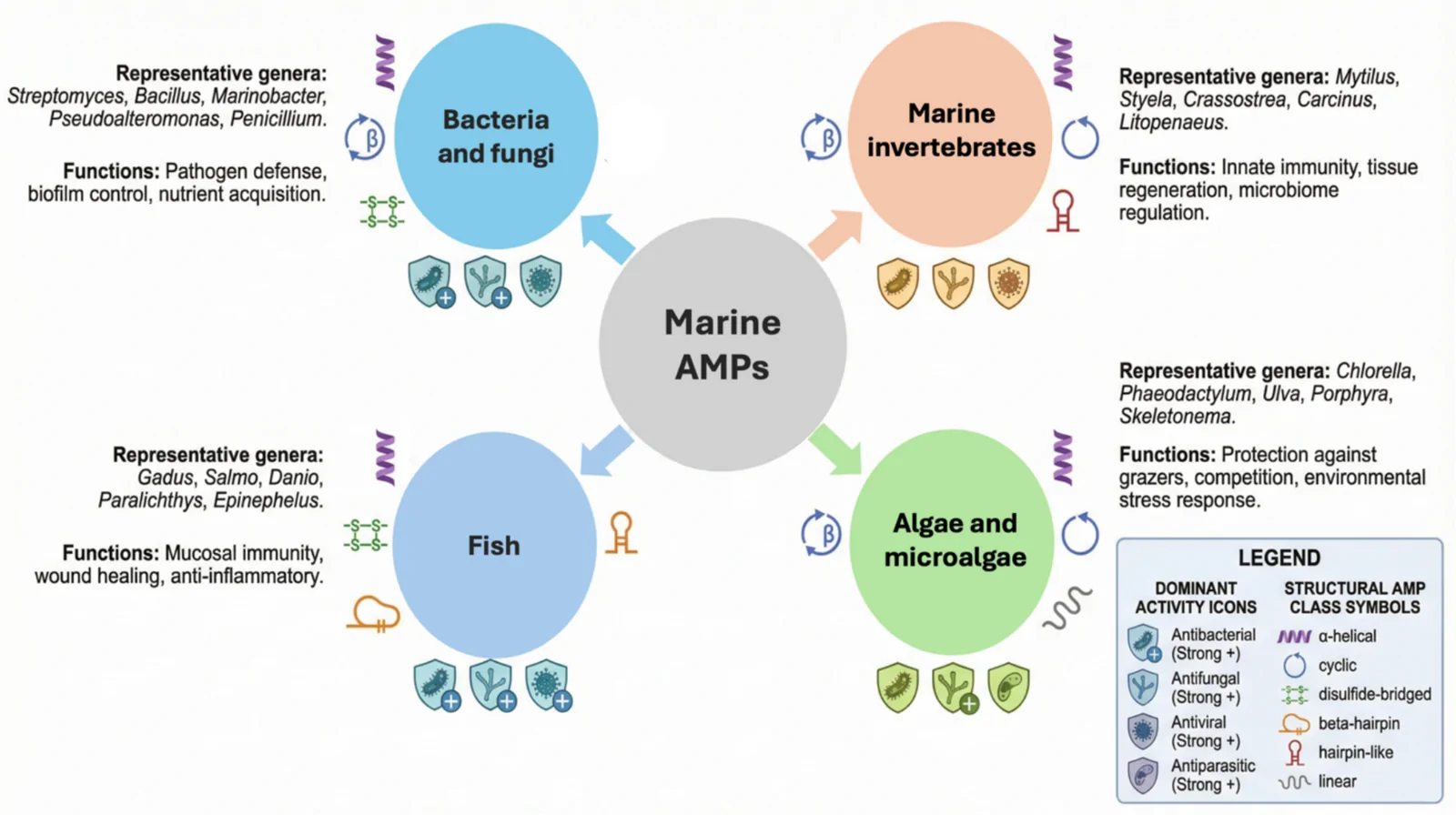
Marine antimicrobial peptides (AMPs) from diverse marine taxa. The figure summarizes major source groups—bacteria and fungi, marine invertebrates, teleost fish, and marine algae and microalgae—and illustrates representative genera, dominant biological activities, and principal structural classes. Structural icons indicate the main AMP families produced by each group (linear α-helical, β-sheet/cysteine-rich, cyclic or depsipeptides including NRPS-derived peptides, and amino-acid-rich or extended peptides), while activity symbols denote predominant antibacterial, antifungal, antiviral, and antiparasitic effects, with “ + ” highlighting particularly strong activities reported for that group. Together, the schematic links taxonomic origin, peptide structure, and functional role in innate immunity, microbiome regulation, ecological competition, and antifouling or allelopathic interactions in marine ecosystems. Activity categories are supported by cited examples in the diversity and classification of marine AMPs

The marine AMPs have been categorized through different classification systems, based on their chemical structure, biological activity, and their aquatic natural source Fig. 1. One of the most widely used classifications is that based on their natural aquatic source. Fish, marine invertebrates, marine plants, and marine microorganisms are the primary sources of marine AMP. Summarizing the overview of the successfully and currently developed marine AMPs linked to their natural marine source is represented in Fig. 2.

Canonical AMP families and their fundamental biological functions can be categorized according to both producing organism and structural class**.** Piscidins and related amphipathic α-helical peptides in marine teleost fish display potent bactericidal and antibiofilm activities and have also been shown to exert immunomodulatory functions within mucosal tissues [63–65]. In crustaceans, penaeidins and crustins represent major AMP families involved in hemolymph-mediated innate defense, exhibiting activity against both Gram-positive and Gram-negative bacteria, with several members additionally demonstrating antifungal properties [12, 66, 67].

In molluscs, particularly bivalves such as mussels, myticins, mytilins, and defensin-like peptides show broad antibacterial activity and play key roles in epithelial and hemolymph immunity [68, 69]. Tunicates produce distinctive AMP families, including styelins and clavanins, which are typically cationic, membrane-active peptides with pronounced antimicrobial potency [39, 53, 56, 70]. Collectively, these canonical AMP families provide well-validated examples linking marine biodiversity to conserved bioactive functions, thereby establishing a robust framework for interpreting organism-level diversity in marine innate immunity, which is depicted in Figs. 1 and 2.

In this section, marine AMPs are defined in a broad, functional context encompassing both canonical ribosomally synthesized innate immune peptides and peptide-based, peptide-derived, or pseudopeptidic marine antimicrobials whose activity is intrinsically linked to amino-acid–based scaffolds. Accordingly, Table 1 includes classical AMPs (e.g., piscidins, defensins, crustins, penaeidins, arenicins) as well as peptide-like marine antimicrobial compounds (e.g., cyclic peptides, depsipeptides, lipopeptides, and hybrid frameworks) that, while not always fitting strict AMP definitions, are well established in the AMP literature. Purely non-peptidic small-molecule antibiotics are included only where historically or structurally relevant. This peptide-centered classification provides a comparative overview of marine antimicrobial diversity and highlights the marine environment as an exceptionally rich source of potent bioactive antimicrobial peptides.

**Table 1:** Marine antimicrobial peptides (AMPs) and peptide-based antimicrobial compounds are classified by source organism, molecular class, and bioactivity and were selected across diverse taxa, including microorganisms, invertebrates, and fish. The exemplars highlight broad structural diversity, functional versatility, and translational potential, underscoring the extraordinary breadth of marine biodiversity as a reservoir for next-generation antimicrobial discovery

Molecule class	Source	Bioactivity	Reference
Marine Microorganisms			
Cyclic dipeptides	*Oceanibulbus indolifex*	Antibacterial	[71, 72]
Andrimid (Pseudopeptides)	*Vibrio* sp.	Antibacterial	[73–78]
Moiramide (Pseudopeptides)	*Vibrio* sp.	Antibacterial	[73–78]
Kahalalides (Depsipeptides)	*Vibrio* sp.	Antifungal	[79–81]
Unnarmicins (Depsipeptides)	*Photobacterium* sp.	Antibacterial	[82, 83]
Ngercheumicins	*Photobacterium* sp.	Antibacterial	[84–88]
Solonamides	*Photobacterium* sp.	Antibacterial	[84–88]
Thiomarinols (Pyrrothine)	*Pseudoalteromonas* sp.	Antibacterial	[89–93]
Cyclopeptides	*Pseudoalteromonas* sp.	Antibacterial	[94–96]
Lipopeptides	*Pseudoalteromonas* sp.	Antibacterial	[94–96]
Massetolides (Cyclic lipopeptides)	*Pseudomonas* sp.	Antimycobacterial	[97, 98]
Myxovalargins (Polyketide/polypeptide hybrid)	*Myxococcus fulvus*	Antibacterial	[99–102]
Althiomycin (Polyketide/polypeptide hybrid)	*Myxococcus fulvus*	Antibacterial	[99, 100, 103–106]
Miuraenamides (Depsipeptide)	*Paraliomyxa miuraensis*	Antifungal	[107, 108]
Arenamide A	*Salinispora arenicola*	Antibacterial	[109]
Asperigillipeptide	*Aspergillus allahabadii; Aspergillus ochraceopetaliformis*	Antibacterial and antifungal	[110–112]
Theopalauamide	*Candidatus Entotheonalla palauensis*	Anticandidal and Antiviral (HIV)	[113, 114]
Chondramides	*Chondromyces crocatus*	Antibacterial	[115]
Microsclerodermins A	*Microscleroderma* sp.	Anticandidal	[116]
Pedeins A and B	*Chondromyces pediculatus*	Antibacterial	[117]
Marine invertebrates			
Clavanins A, B, C, D, and E	*Styela clava*	Antibacterial	[70]
Dicynthaurin	*Halocynthia aurantium*	Antibacterial	[118]
Halocyntin	*Halocythnia papillosa*	Antibacterial	[119]
Hedistin	*Nereis diversicolor*	Antibacterial	[38]
Papillosin	*Halocynthia papillosa*	Antibacterial	[119]
Epinecidin-1	*Epinephelus coioides*	Antibacterial, anticancer, wound healing	[120]
Octominin	*Octopus minor*	Antibacterial, anticandidal, and Antibiofilm	[121–125]
Octopromycin	*Octopus minor*	Antibacterial and antibiofilm	[126]
Octoprohibitin	*Octopus minor*	Antibacterial	[127]
Styelins A, B, C, D, and E	*Styela clava*	Antibacterial	[70, 128]
Arasin 1	*Hyas araneus*	Antibacterial	[129, 130]
Callinectin	*Callinectes sapidus*	Antibacterial	[131, 132]
Hyastatin	*Hyas araneus*	Antibacterial and anticandidal	[25, 26]
Arenicins 1, 2, and 3	*Arenicola marina*	Antibacterial	[133–135]
Aurelin	*Aurelia aurita*	Antibacterial	[136, 137]
Crustins types I, II, and III	*Carcinus maenas;* *Portunus trituberculatus*	Antibacterial	[138, 139]
Damicornin	*Pocillopora damicornis*	Antibacterial	[140]
Hepcidins	*Sparus aurata*	Antibacterial	[141]
MCdef	*Ruditapes philippinarum*	Antibacterial	[142]
Myticins A, B, and C	*Mytilus galloprovincialis*	Antibacterial	[143]
Mylitin	*Mytilus galloprovincialis*	Antibacterial	[69, 144]
Penaeidins 1, 2, and 3	*Penaeus vannamei*	Antibacterial	[145]
Poliphemusin	*Limulus polyphemus*	Antibacterial	[146, 147]
Strongylocins 1 and 2	*Strongylocentrotus droebachiensis*	Antibacterial	[35]
Tachyplesins I, II, and III	*Tachypleus tridentatus*	Antibacterial	[55, 146–148]
Discodermin A	*Discodermia kiiensis*	Antibacterial	[149–151]
Theonegramide	*Theonella swinhoei*	Anticandidal	[152]
Theonellamide G	*Theonella swinhoei*	Anticandidal	[153]
Cyclolithistide A	*Theonella swinhoei*	Antibacterial, antifungal, and antiprotozoal	[154]
Koshikamides F and H	*Theonella* sp.	Antiviral (HIV)	[155, 156]
Halicylindramide D and E	*Halichondria cylindrata*	Antifungal (*M. ramannian*)	[157–159]
Aciculitin A	*Aciculites orientalis*	Antiviral (HIV)	[160]
Callipeltin A	*Callipeltin sp.;* *Latrunculia sp.*	Antiviral (HIV) and anticandidal	[161]
Neamphamide A	*Neamphius huxleyi*	Antiviral and antimycobacterial	[162]
Homophymine A	*Homophymia* sp.	Antiviral (HIV)	[163]
Theopapuamide A, B, and C	*Siliquariaspongia mirabilis*	Anticandidal and antiviral (HIV)	[164–166]
Celebeside A	*Siliquariaspongia mirabilis*	Antimycobacterial	[167]
Mirabamide A, C, and D	*Siliquariaspongia mirabilis*	Antiviral (HIV), anticandidal, and antibacterial	[168–170]
Mirabamide H	*Stelletta clavosa*	Antiviral (HIV), anticandidal, and antibacterial	[171]
Jasplakinolide (Jaspamide)	*Jaspis johnstoni*	Antibacterial	[172]
Stellettapeptin A and B	*Stelletta clavosa*	Antiviral (HIV)	[21, 173]
Callyarein A and B	*Callyspongia aerizusa*	Antimycobacterial	[174, 175]
Bac-like	*Carcinus maenas*	Antibacterial	[176]
Callinectin	*Callinectes sapidus*	Antibacterial	[131, 132]
Astacidin-2	*Pacifastacus leniusculus*	Antibacterial	[177]
Armadillidin	*Armadillidium vulgare*	Antibacterial and antifungal (mold)	[178]
Homarin (CAP-1)	*Homarus americanus*	Antibacterial and antiprotozoal	[179]
Defensins	*Panulirus japonicus*	Antibacterial, antifungal, and antiviral	[180]
Anti-LPS factor (ALF)	*Metapenaeus dobsoni; Penaeus* sp*p.; Neocaridina heteropoda; Macrobrachium* spp.; *Palaemon carinicauda; Homarus americanus; Cherax quadricarinatus; Pacifastacus leniusculus; Procambarus clarkii; Eriocheir sinensi; Chionoecetes opilio; Charybdis* spp.; *Portunus* spp.; *Scylla* spp.	Antibacterial	[181–197]
Scygonadin	*Scylla serrata*	Antibacterial and antiviral	[198]
Scylla serrata antimicrobial protein (SSAP)	*Scylla serrata*	Antibacterial	[199]
Penaeidin	*Penaeus monoceros*	Antibacterial	[200]
Crustin	*Artemia salina; Penaeus* spp.;* Neocaridina heteropoda; Macrobrachium nipponense; Pandalus japonicas; Rimicaris exoculata; Panulirus japonicus* *Homarus* spp.;* Cherax quadricarinatus; Pacifastacus leniusculus; Paralithodes camtschaticus; Hyas araneus; Portunus pelagicus; Scylla spp.*	Antibacterial	[25–28, 54, 179–181, 190, 196, 199–211]
Hyastatin	*Hyas araneus;* *Cionoecetes opilio;* *Portunus trituberculatus*	Antibacterial	[212]
SpHyastatin	*Scylla paramamosain*	Antibacterial	[212]
Arasin	*Hyas araneus*	Antibacterial	[130]
Stylicin	*Penaeus* spp.;* Penaeus japonicus*	Antibacterial (Gram negative) and antifungal (mold)	[200]
Pellino-1-derived cationic antimicrobial prawn peptide	*Macrobrachium rosenbergii*	Antibacterial	[213]
Histones or histone-derived peptides	*Penaeus* spp.	Antibacterial	[192, 214]
Hemocyanin-derived peptides	*Penaeus* spp*.*;* Pacifastacus leniusculus; Procambarus clarkii*	Antibacterial	[13, 215, 216]
Spgly-amp	*Scylla paramamosain*	Antibacterial	[217]
Scyreprocin	*Scylla paramamosain*	Antibacterial, antifungal (mold), and antibiofilm	[218]
Neosiphoniamolide	*Neosiphonia supersets*	Antibacterial	[219]
Ecteinascidin	*Reniera* sp.	antineoplastic, antihypertensive, antioxidant, antimicrobial, anticytotoxic, antibacterial, antifungal, insecticidal, anti-HIV and antiparasitic, antimalarial, antitrypanosomal, anticancer	[220]
Fishes			
Misgurin	*Misgurnus anguillicaudatus*	Antibacterial and anticandidal	[221]
Hagfish Intestinal AMP	*Eptatretus burgeri*	Antibacterial and anticandidal	[222]
Chrysophsins	*Chrysophrys major*	Antibacterial	[223]
Moronecidin	*Morone chrysops;* *Menticirrhus saxatilis*	Antibacterial	[65, 224]
Piscidins 1, 2, 3, 4, and 5	*Oreochromis niloticus*	Antibacterial	[225]
Myxinidin	*Myxine glutinosa*	Antibacterial and anticandidal	[226–228]
Parasin 1	*Parasilurus asotus*	Antibacterial	[229, 230]
Pleurocidin	*Pleuronectes americanus*	Antibacterial	[231, 232]
Astacidin 1 and 2	*Pacifastacus leniusculus*	Antibacterial	[177, 215]
Chrysophsin 1, 2 and 3	*Chrysophrys major*	Antibacterial	[223, 233, 234]

The table includes both canonical AMPs and peptide-derived or pseudopeptidic marine antimicrobial compounds to provide a comprehensive peptide-centered overview.

## Technological Advances in Marine AMP Discovery

The biodiscovery pipeline has traditionally used a bioassay-guided approach to isolate bioactive natural compounds from natural sources. This approach demonstrated a high percentage of success in detecting these chemicals, but it also had several shortcomings. This method's main flaw is its strong possibility of isolating substances that are already well-known [235].

### Omics-Guided Discovery

High-resolution "omics" technologies—such as genomes, transcriptomics, proteomics, and metabolomics—are revolutionizing the discovery process for marine AMPs by facilitating the swift identification of biosynthetic capabilities directly from environmental or organism-specific datasets. Recent multi-omics investigations [37, 236–238] illustrate that the integration of genome sequencing, transcriptomic profiling, and peptide-centric proteomics can uncover concealed AMP families, clarify post-translational modifications, and associate biosynthetic gene clusters with bioactive phenotypes.

These advancements are bolstered by progress in computational mining instruments and high-throughput sequencing. Long-read sequencing, metagenome-assembled genome (MAG) reconstruction, and deep-learning peptide-prediction platforms facilitate the identification of AMP-encoding biosynthetic gene clusters in uncultured marine microorganisms [46, 239, 240]. Contemporary pipelines increasingly incorporate predictive genome-mining instruments, including antibiotics and secondary metabolite analysis shell (antiSMASH) and bacteriocin genome mining tool (BAGEL), alongside hidden Markov model (HMM)-based motif searches and probe-guided contig scanning to elucidate atypical or fragmented gene clusters. These methodologies collectively establish genome mining as a pivotal tool in marine natural-product biodiscovery [235, 241–246].

Crucially, peptidomics now serves as the principal experimental validation layer in this workflow. While untargeted metabolomics has a significant practical constraint is the selection of extraction conditions that can solubilize a chemically varied array of metabolites while preserving the integrity of the overall metabolome. Medium-polarity solvents like methanol, ethanol, or acetone are frequently utilized for wet or lyophilized tissues, and solid-phase extraction (SPE) is regularly employed to mitigate salt interference and improve chromatographic resolution [247–250].

For AMPs, discovery is predominantly guided by sequence and peptide-spectrum analysis. Transcriptomic profiling (RNA-seq) facilitates the swift identification of AMP precursors and their regulated expression in response to microbial challenges, whereas peptidomics (LC–MS/MS) confirms mature peptide products, post-translational modifications, and variations in abundance. These methodologies are enhanced by genome mining of precursor genes and antimicrobial motif signatures to identify AMP candidates, even when peptide output from biomass is minimal [251].

While initial marine metabolomics predominantly utilized nuclear magnetic resonance (NMR) spectroscopy [252], ongoing technological advancements have established mass spectrometry—especially ultra-high performance liquid chromatography–tandem mass spectrometry (UHPLC-MS/MS)—as the preeminent method for AMP discovery, owing to its sensitivity, rapid throughput, and extensive metabolite coverage. MS/MS-based de novo peptide sequencing and global natural product social (GNPS) molecular networking have become essential for dereplication, structural annotation, and grouping of analogous peptide ions. Here, GNPS molecular networking is used within a peptidogenomics framework to cluster related peptide MS/MS spectra, accelerate dereplication, and facilitate linkage to candidate AMP precursor genes.

Recent advancements in mass spectrometry hardware, high-resolution analyzers, and computational dereplication have enhanced mass accuracy to the sub-ppm level and reinforced connections between molecular ions and biosynthetic gene clusters [47, 253–256]. AI-assisted sequence–function modelling has become a pivotal component in AMP prioritization, enabling in silico predictions of antimicrobial efficacy, structural integrity, activity spectrum, and haemolytic risk directly from MS/MS-derived peptide sequences. This integration significantly decreases experimental attrition and concentrates subsequent functional screening on the most promising candidates.

NMR maintains complementary significance by facilitating direct structural elucidation and quantification using 1D 1H-NMR without the need for derivatization, while 2D experiments are employed when intricate mixtures necessitate enhanced resolution [257]. Its repeatability and non-destructive characteristics render it especially appropriate for comparative or time-series metabolomics. Together, the convergence of genome mining, peptidomics, high-throughput sequencing, and AI-driven analytics is reshaping marine AMP discovery into a streamlined, peptide-first pipeline with direct translational relevance and expediting the identification of marine AMPs and broadening the available chemical landscape for biotechnological innovation.

### Bioinformatics and Genome Mining

Due to the fact that most AMPs, such as defensins and cathelicidins, often surpass approximately 3 kDa and necessitate peptide-centric methodologies, metabolomics is typically not a principal discovery instrument for AMPs, with the exception of specific low-molecular-weight cyclic peptides or peptide-derived metabolites. Biosynthetic gene clusters (BGCs) that govern the synthesis of natural products often remain inactive under conventional laboratory conditions, leading to a restricted identification of their corresponding metabolites [241]. Proteomic and transcriptomic analysis ascertain the active expression of these BGCs, with transcriptomics offering a direct method to differentiate between silent and inducible clusters without necessitating prior genomic information [243]. Expression-based methodologies elucidate active pathways, whereas high-throughput metabolomics enhances this by identifying downstream products from both established and novel biosynthetic pathways, facilitating swift detection of chemical diversity at scales that surpass conventional bioassay-guided processes [258].

Contemporary metabolomics emphasizes the thorough characterization of low-molecular-weight molecules (≤ 1500 Da), many of which play ecological defense roles and enhance competitive fitness in marine animals. Its ability to identify various molecular species significantly aids in the discovery, quantification, and structural characterization of new metabolites, therefore facilitating purification and functional evaluation.

Nevertheless, metabolomics alone cannot elucidate the complete biosynthetic capacity of marine microorganisms, as numerous AMPs stem from cryptic or inadequately expressed biosynthetic gene clusters. Consequently, genome-driven discovery processes are progressively augmenting metabolomic screening. Modern methodologies—such as motif-guided HMMER3 searches for conserved AMP signatures [259], deep-learning frameworks for predicting atypical or fragmented BGCs [37, 260], probe-guided contig scanning for hallmark precursor motifs [261], and MAG reconstruction that retrieves nearly complete genomic bins from environmental data [262, 263]—facilitate the systematic identification of cryptic AMP-encoding loci, even in the absence of detectable metabolite production. These integrative methodologies enhance accessibility to biosynthetic domains that would otherwise remain undetected in solely metabolomics-focused analyses.

Notwithstanding the intricacies associated with untargeted MS- or NMR-based profiling, thorough metabolome characterization is necessary. Metabolomics facilitates the structural description and measurement of newly identified molecules, thereby confirming genome-derived predictions and connecting genetic potential to chemical reality.

### High-Throughput Screening and Synthetic Biology

Marine natural-product (MNP) databases are essential for dereplicating raw extracts by facilitating the fast comparison of newly identified metabolites with previously characterized structures. Effective dereplication necessitates the use of structural characteristics, including molecular mass, elemental composition, MS fragmentation patterns, and, when accessible, NMR spectrum data. Wolfender et al. [264] present a revised summary of specialized marine databases,nonetheless, only a limited number provide extensive and biologically searchable content, and even fewer have over 50 annotated marine natural products. Generic metabolomics databases, exemplified by the Dictionary of Marine Natural Products [265], provide comprehensive taxonomic indexing,nonetheless, they are still deficient, especially for under-researched taxa like tunicates, molluscs, and echinoderms. The MetaboLights library enumerates more than 130 sponge-derived chemicals but lacks vetted entries for numerous other significant marine invertebrate taxa.

The restricted scope of current MNP databases, coupled with variability in data formats, poses a difficulty for effective compound identification. Resources for structure prediction that produce theoretical MS or NMR spectra [266] can partially mitigate these deficiencies, while databases that amalgamate experimental spectral data—alongside annotations of biological origin, molecular targets, and recorded bioactivities—significantly enhance the dependability of dereplication processes. AMP validation and annotation are most facilitated by specialized peptide resources rather than metabolite-focused databases. Databases like dbAMP 2.0, APD3, CAMP3, and DRAMP 4.0 offer curated AMP sequences along with activity metadata, target organism details, physicochemical attributes, structural annotations, and, in certain instances, toxicity or haemolysis data. Incorporating these resources into discovery pathways facilitates standardized annotation, minimizes false positives from generic gene mining, and enhances comparative benchmarking of newly found marine AMP candidates.

Concurrent with database building, recent biotechnological advancements have significantly expedited the progression from marine AMP discovery to functional assessment. While conventional bioassay-guided isolation produces limited peptide amounts via labour-intensive processes, contemporary methods facilitate identification without necessitating significant extraction. Genome mining, transcriptome profiling, and proteomic analyses can directly identify AMP-encoding genes from biological material, including unculturable species. Upon identification of candidate sequences, synthetic biology and recombinant expression technologies provide scalable manufacturing for subsequent testing.

**Fig. 3:**
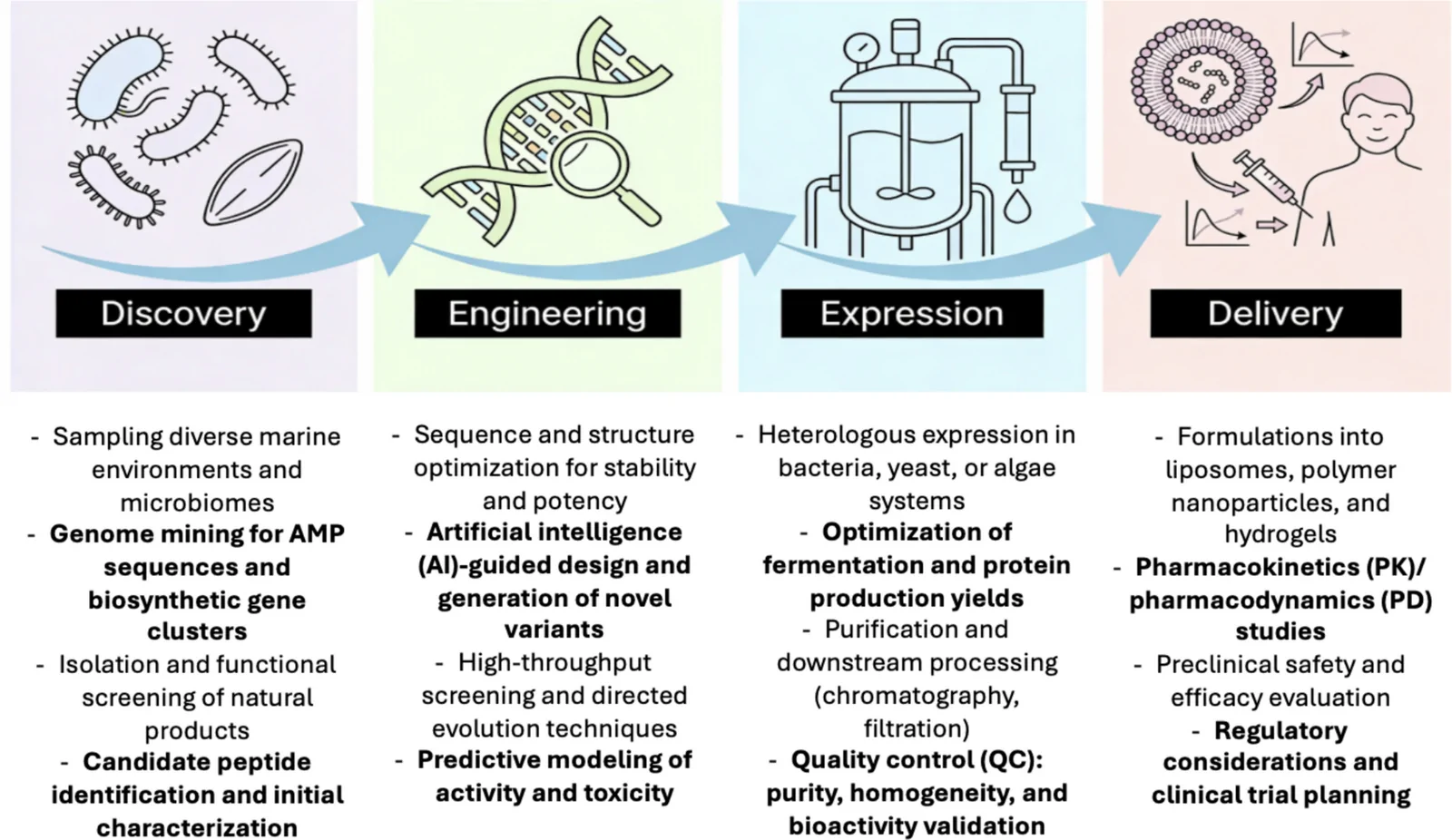
Translation pipeline for marine antimicrobial peptides (AMPs) from discovery to clinical application. The graphic outlines the sequential phases required to advance marine AMPs into clinical prospects, beginning with the discovery of marine biodiversity, genome mining, and metagenomic datasets, followed by mass spectrometry/NMR dereplication and antimicrobial/antibiofilm bioactivity screening. The engineering phase involves optimizing sequence and structure, incorporating AI-assisted design, and introducing stability-enhancing characteristics (such as D-amino acids, cyclization, and stapling), while also adjusting potency, selectivity, and anticipated toxicity. During the expression phase, optimized AMP constructs are generated in heterologous hosts, including *Escherichia coli*, *Pichia pastoris*, or insect cell systems, utilizing suitable fusion tags and secretion signals, subsequently undergoing purification (e.g., HIC, RP HPLC) and quality assessment for identity, endotoxin levels, and biological activity. The delivery phase emphasises sophisticated formulations—such as liposomes, polymer nanoparticles, and hydrogels—intended to target biofilms and mucosal surfaces, while considering pharmacokinetics/pharmacodynamics (PK/PD), stability in intricate matrices, and regulatory and manufacturability criteria for clinical application

Moreover, computer modelling and AI-driven prediction platforms can assess antibacterial efficacy, stability, potential toxicity, and physicochemical characteristics prior to laboratory confirmation. The integration of dereplication technologies, curated databases, genome-driven discovery, and synthetic production markedly accelerates the development process, promoting the swift progression of marine AMPs for biotechnological and medicinal uses, as illustrated in Fig. 3.

## Extraction, Purification, and Synthetic Production

The direct extraction of bioactive peptides from marine organisms is inherently difficult due to significant variability in seasonal availability, population density, geographic distribution, and ecological habitat, which collectively restrict biomass accessibility and reproducibility [267–269]. Conventional antimicrobial discovery procedures commence with specimen collection, proceed to tissue isolation, mechanical trituration, or cellular lysis, and involve the following clarification processes, such as extraction, concentration, precipitation, centrifugation, and membrane filtration. Chromatographic methods—including size-exclusion chromatography (SEC), ion-exchange chromatography (IEC), and solid-phase extraction (SPE)—are utilized to eliminate inorganic salts, lipids, and high-molecular-weight impurities before bioactivity assessment [68, 270]. Nonetheless, the direct extraction of AMPs from lyophilized marine tissues is generally ineffective due to their low natural abundance, swift breakdown, and interference from intricate biological matrices.

As a result, modern AMP discovery pipelines have transitioned to sequence-driven methodologies, emphasizing: (i) de novo identification of AMP precursors via transcriptome mining (RNA-seq) or targeted PCR techniques; (ii) molecular validation and structural characterization through peptidomics (LC–MS/MS) combined with differential expression analyses (e.g., qPCR) in response to microbial or immune stimulation; and (iii) spatial localization through immunohistochemistry when functional context is necessary. For downstream functional assessment and standardized bioassays, AMPs are predominantly synthesized by solid-phase peptide synthesis (SPPS) or heterologous recombinant expression methods, guaranteeing consistent yields, exact structural control, and stringent quality assurance. These advancements have transformed marine AMP discovery into a genome-informed, peptide-centric process, addressing the practical constraints of direct extraction and facilitating scalable biotechnological development.

### Traditional Isolation Methods

#### Chromatography

Using acetic acid extraction, C18 solid-phase extraction, reversed-phase high-performance liquid chromatography (RP-HPLC), size-exclusion chromatography, and two further purification rounds of RP-HPLC [267, 271–275]. Comparable multi-step purification procedures are used for other marine AMPs,the details of these procedures are decided by the AMPs themselves. Since the presence of bioactive peptides is assessed at each step, the method of isolating and purifying AMPs is termed "bioassay-guided purification".

#### Expression-Driven Discovery

Alternative discovery methodologies utilize inducible AMP expression by subjecting the source organism to bacterial or immunological challenges, thereby activating innate defense pathways and augmenting AMP transcription. Preliminary research indicated that pathogen exposure can enhance AMP expression profiles, aiding in the subsequent discovery of immune-responsive peptides. Based on this idea, constructing cDNA libraries from immune-stimulated tissues has been a prevalent method for AMP discovery. Total RNA is isolated from challenged organisms, reverse-transcribed into cDNA, and screened for AMP-encoding transcripts in this procedure. Primer-specific amplification and targeted sequencing facilitate the swift identification of open reading frames associated with AMP precursors, even at low peptide abundance [251]. These expression-driven techniques are predominantly discovery-oriented, aimed at identifying novel AMP genes and precursor structures, while large-scale peptide manufacture and functional validation are generally accomplished by recombinant or synthetic platforms discussed in later sections. Recently, inducible expression data have been included in transcriptome and proteomic processes, facilitating the prioritization of high-confidence AMP candidates before synthetic or recombinant synthesis.

### Chemical Synthesis and Peptide Engineering

The chemical synthesis of marine AMPs has recently been investigated as a solution to the scarcity of source organisms in large quantities. Total chemical synthesis of peptides is preferable to isolation from marine species since it preserves an ecosystem's biodiversity [276]. A two-step solid phase/solution synthesis of bioactive extremely efficient anti-lipopolysaccharide factors (ALFs), a type of extremely efficient AMP [277, 278].

### Recombinant Production

Recent advancements in sequencing technology and multi-omics platforms have significantly expedited the identification of marine AMPs by facilitating swift, economical access to extensive genomic and biochemical information. Integrated methodologies that amalgamate transcriptomics, proteomics, metabolomics, and data-mining frameworks—augmented by high-resolution analytical instruments like LC–MS/MS—facilitate the accurate identification of novel AMP candidates from various marine organisms [279, 280]. These strategies diminish dependence on conventional extraction-based discoveries and significantly expand the taxonomic diversity available for AMP characterization. 

In conjunction with these technological advancements, ethical and ecological considerations are paramount in marine bioprospecting. Numerous marine invertebrates generate trace amounts of AMPs, and extensive extraction for compound separation can jeopardise fragile ecosystems—an issue commonly referred to as the “supply problem” in marine natural products [281–283]. International access-and-benefit-sharing systems, exemplified by the Convention on Biological Diversity (CBD), underscore the necessity for legally compliant and environmentally sustainable sourcing [284, 285].

Synthetic chemistry and recombinant expression systems tackle these problems by facilitating laboratory-based synthesis of marine peptides without the need for biomass extraction. Chemical synthesis enables meticulous regulation of peptide architecture and facilitates the production of stable analogues with enhanced pharmacological characteristics [286]. Concurrently, recombinant expression in heterologous hosts—such as *Escherichia coli*, *Pichia pastoris*, and insect cell systems—offers scalable and economical methods for generating native or engineered AMP variants with minimal ecological repercussions [287–293].

By detaching AMP supply chains from marine gathering, synthetic and recombinant methods guarantee sustainability and production dependability, facilitating industrial, biological, and therapeutic advancements [294, 295]. These initiatives integrate marine biotechnology with overarching conservation goals and are crucial for the ethical and responsible development of AMP-based medicines.

## Mechanisms of Action and Functional Roles of Marine AMPs

AMPs initially interact biologically with the host cell membrane's phospholipids. Following is a list of the five distinct phases that make up AMP-host interaction: direct contact with target cells through biochemical or biophysical affinity (e.g., electrostatic or hydrophobic interactions), structural modification of the target membrane (formation into a helix or barrel structure), accumulation up to an active stoichiometric level, disruption of the target membrane through permeabilization or depolarization, or causing any other direct or indirect abnormality in its function that can be temporary or permanent [296, 297]. Following the bacterial cell-AMP molecule interaction, many AMPs can follow one of the primary modes of action for killing microbes [298] as follows:

### Mechanism of Action of Marine AMPs

#### Classic AMP Model

##### Membrane Dysfunction

The cell membrane is essential for sustaining electrochemical gradients and facilitating membrane-associated electron transport; any impairment, such as ion or metabolite leakage, leads to depolarization and the failure of respiratory function, ultimately resulting in microbial death either directly or via subsequent metabolic failure. Numerous established mechanisms elucidate the manner in which AMPs destabilize the lipid bilayer of their targets [298–302]. Aurelin, a cationic AMP derived from the jellyfish *Aurelia aurita*, possesses structural characteristics akin to defensins and potassium channel-blocking toxins, suggesting its role in disrupting membrane ion channel functionality and microbial electrochemical gradients [136]. This form of AMP-induced disruption undermines both membrane integrity and the energy equilibrium vital for microbial viability (Table 2).

**Table 2:** Representative marine antimicrobial peptides (AMPs) and their membrane-disruptive mechanisms

Mechanism	Representative Marine AMPs	Source Organism	Structural Class	Mechanistic Notes	Reference
Carpet Model	Myticin C	*Mytilus galloprovincialis* (mussel)	Cys-rich peptide	Spreads across membrane surface; detergent-like disruption	[303]
	Halocyntin	*Halocynthia aurantium* (tunicate)	α-helical peptide	Surface accumulation → bilayer solubilization	[304]
	Aurelin	*Aurelia aurita* (jellyfish)	Defensin-like peptide	Disrupts ionic flux and electrochemical gradients	[136]
Toroidal (Peptide–Lipid) Pore	Piscidin-1	Teleost fish	Amphipathic α-helix	Deep insertion and leaflet curvature → toroidal pore formation	[224]
	Tachyplesin I	Horseshoe crab	β-sheet, disulfide stabilized	Mixed peptide–lipid pore formation; strong headgroup binding	[55]
	Penaeidin-3	Shrimp	Proline-rich + cysteine-rich domains	Curvature induction leading to toroidal channels	[145]
Barrel-Stave Pore	Pardaxin	*Pardachirus marmoratus* (flatfish)	Amphipathic α-helix	Classic barrel-stave pore; peptide oligomers form pore wall	[305]
Membrane Thinning / Non-Pore Disruption	Clavanin A	*Styela clava* (tunicate)	Histidine-rich α-helix	pH-dependent insertion; bilayer softening & thinning	[306]
	Dicentracin	Sea bream	α-helical peptide	Disrupts bilayer thickness and mechanical stability	[64]
Intracellular Targeting (after mild permeabilization)	Hepcidin	Teleost fish	Cys-rich β-sheet	Weak pore formation; metal sequestration	[307]
	NK-lysins	Teleost fish	α-helix	Membrane disruption followed by intracellular killing	[308]

##### Carpet Model

AMPs initially adhere electrostatically to negatively charged bacterial membranes and align parallel to the bilayer surface [305, 309]. As surface coverage escalates, they aggregate into a carpet-like configuration over the phospholipid headgroups [161, 310, 311]. Upon attaining a critical concentration, the peptide-lipid complex disrupts the bilayer in a detergent-like fashion, solubilizing membrane regions and forming micelle-like aggregates, resulting in membrane permeabilization and integrity loss [161, 301, 302, 309, 312, 313]. Marine-derived AMPs exemplify this method clearly: myticin C, derived from the mussel *Mytilus galloprovincialis*, demonstrates robust cationic surface adhesion and spreading consistent with the carpet model [303], whereas Halocyntin from the tunicate *Halocynthia aurantium* similarly aggregates on microbial membranes and prompts detergent-like bilayer disruption [304]. Collectively, these peptides illustrate the efficacy of marine invertebrate AMPs in destabilizing lipid structures via carpet-mediated membrane disruption (Table 2).

##### Toroidal or Wormhole Model

AMPs induce curvature in lipid leaflets, resulting in a pore that is bordered by both peptide and phospholipid, characteristic of a toroidal (peptide–lipid) pore [314–317]. Upon adequate surface accumulation, peptides reorient, and a central water channel curves through the bilayer, developing into a transmembrane pore once a critical peptide-to-lipid ratio is surpassed [314, 316–319]. Marine AMPs exemplify this mechanism: piscidin-1, derived from teleost fish, creates mixed peptide-lipid pores exhibiting a toroidal architecture [224], whereas tachyplesin I, a disulfide-stabilized β-sheet peptide from the horseshoe crab, promotes bending of phospholipid headgroups indicative of toroidal pore formation [55] (Table 2).

##### Barrel-Stave Model

Peptides of sufficient length to traverse the bilayer insert as α-helices almost perpendicular to the membrane and oligomerize to create a barrel-stave pore [320, 321]. In this configuration, each helix orients its hydrophilic surface towards the aqueous lumen of the channel, while hydrophobic residues are directed outward to interact with the hydrocarbon core of the phospholipid bilayer, consequently, the bundle creates a transmembrane, water-filled channel exhibiting stepwise conductance that correlates with variations in oligomer quantity [161, 317, 322]. Direct structural and biophysical evidence for barrel-stave pores has been most clearly demonstrated for alamethicin, whose oligomeric helical bundles have been elucidated using neutron and X-ray scattering/diffraction [323, 324]. Pardaxin, a peptide produced from marine sources, originates from flatfish. *Pardachirus marmoratus* serves as a quintessential example of this model: it integrates as a slanted α-helix, forms multimeric transmembrane bundles, and generates voltage-dependent, distinct conductance steps typical of barrel-stave pores [305]. Likewise, some marine-derived magainin analogues demonstrate helical insertion and oligomerization characteristics that are frequently regarded as intermediate between barrel-stave and toroidal mechanisms, while nevertheless exhibiting typical barrel-like channel creation under particular membrane conditions [325]. Collectively, these peptides demonstrate the self-organization of amphipathic helices into rigid transmembrane bundles that serve as distinct aqueous apertures (Table 2).

#### Non-Membrane Role

##### Extracellular Biopolymer Synthesis Inhibition

An important non-lytic mode of action of AMPs is the inhibition of essential extracellular macromolecular biosynthetic processes [326, 327], particularly those involved in cell envelope biogenesis. These include disruption or suppression of peptidoglycan (PGN) assembly in Gram-positive bacteria and interference with lipopolysaccharide (LPS) biogenesis and outer membrane (OM) integrity in Gram-negative bacteria, thereby compromising envelope homeostasis and sensitizing cells to downstream killing mechanisms [328–331].

##### Intracellular Function Inhibition

Beyond envelope-associated effects, several AMPs exert intracellular activities after translocation, contributing to bacteriostasis or bactericidal outcomes through mechanisms distinct from membrane disruption. These include interactions with ATP and ATP-dependent enzymes [332, 333], as well as inhibition of core intracellular processes such as protein synthesis, nucleic acid synthesis, and enzyme function [334–342]. This mechanistic duality helps explain both broad-spectrum efficacy and certain translational challenges (e.g., toxicity, hemolysis, and protease sensitivity), while also creating selection pressure for resistance strategies such as surface charge modification and biofilm shielding. Mechanistic resolution, therefore, directly informs peptide engineering—biasing activity toward pathogen-selective targets can reduce hemolysis, improve pharmacokinetics (PK), and enhance therapeutic index.

##### DNA Binding/Lysis and Ribosomal Inhibition

An expanding corpus of evidence suggests that a specific category of AMPs—especially proline-rich antimicrobial peptides (PrAMPs) and select cationic β-sheet peptides—primarily exert antibacterial effects via intracellular target interaction, particularly ribosomal inhibition and nucleic-acid binding, rather than through direct membrane lysis [332, 341]. PrAMPs are commonly internalized through bacterial inner-membrane transport systems, such as SbmA/BacA-type importers, and then associate with the 70S ribosome, typically targeting the polypeptide escape tunnel or nearby functional domains. This interaction inhibits translation initiation or elongation, leading to the swift cessation of protein synthesis and either bacteriostasis or cell death [334, 340, 341]. These non-lytic processes are now structurally and mechanistically substantiated and are increasingly acknowledged as a significant paradigm of AMPs beyond membrane disruption [339].

Marine instances highlight the significance of these intracellular pathways. The dolphin-derived proline-rich peptide Tur1A inhibits bacterial protein synthesis by directly binding to ribosomes and selectively obstructing the transition from translation initiation to elongation, exemplifying a typical PrAMP-like intracellular mechanism. Proline/arginine-rich peptides, such as arasin-1 derived from the spider crab *Hyas araneus*, are often examined within the PrAMP framework in marine invertebrates and serve as marine scaffolds with significant potential for enhancement aimed at ribosome-targeting, non-lytic antibacterial activity [130, 341].

Simultaneously, some marine AMPs demonstrate direct binding to nucleic acids, enhancing antimicrobial efficacy by disrupting transcriptional and replicative mechanisms and initiating subsequent stress responses. The horseshoe crab peptide tachyplesin-derived Tatritin has been documented to bind DNA and interact with LPS and chitin, indicating that nucleic-acid engagement is a significant non-membrane mechanism in marine AMP biology [55]. The DNA binding of β-sheet AMPs from marine invertebrates, such as tachyplesin-class peptides, has been consistently observed and is a crucial molecular theme for elucidating non-lytic AMP activity, where peptide sequence and structure facilitate nucleic acid affinity [333].

These non-membrane processes collectively broaden the molecular framework of marine AMPs beyond traditional membrane disruption. They also offer practical engineering guidance: ribosome-targeting PrAMP scaffolds can be refined for cellular absorption, target contact, and diminished host toxicity, while DNA-binding peptides can be systematically adjusted to enhance pathogen specificity and reduce off-target effects. This endorses the classification of ribosomal inhibition and nucleic-acid targeting as separate, therapeutically significant non-lytic processes in the discovery and engineering of marine AMPs.

#### Biotechnological Relevance: Engineering Peptides for Enhanced Mechanism Selectivity

A key benefit of AMPs is their mechanistic diversity, which includes membrane disruption, intracellular target interference, and immunological regulation, thus establishing a robust basis for rational optimization. Although initial structure–activity relationship (SAR) investigations highlighted the significance of amphipathicity, charge distribution, and secondary structure, recent advancements in AMP engineering from 2021 to 2025 are predominantly propelled by artificial intelligence (AI)–assisted design, molecular dynamics (MD) simulations, and integrated computational–experimental methodologies.

Models based on deep learning (DL), such as convolutional neural networks, recurrent architectures, and transformer frameworks, have been trained on growing AMP repositories to concurrently predict antimicrobial potency, membrane selectivity, haemolytic risk, protease resistance, and physicochemical stability directly from peptide sequences. From 2021 to 2025, numerous studies indicated that AI-driven sequence generation surpasses conventional rational design by navigating extensive combinatorial spaces while maintaining biological plausibility, facilitating the de novo design and optimization of AMP analogues with improved therapeutic indices [28, 37, 237, 343].

Simultaneously, contemporary atomistic and coarse-grained MD simulations have evolved from mere descriptive assessments to become predictive engineering instruments. Recent MD investigations have elucidated peptide–membrane insertion dynamics, lipid selectivity, oligomerization behaviour, and pore-forming mechanisms at near-physiological resolution, facilitating the exact modulation of helix stability, β-sheet rigidity, and charge topology before synthesis. The MD-guided optimization of marine AMPs, specifically epinecidin-1 and piscidin-derived analogues, has produced variations exhibiting improved bacterial membrane selectivity and decreased erythrocyte lysis [46, 47].

AI–MD hybrid workflows now define the forefront of AMP engineering. These pipelines combine machine learning (ML)–based sequence prioritization with MD-validated mechanistic filtering, subsequently leading to targeted synthesis and biological validation. From 2023 to 2025, these techniques have produced AMP candidates with enhanced serum stability, extended in vivo half-life, and decreased immunotoxicity, as seen in mouse infection and PK models [238, 343]. Recent computational developments have together turned marine AMPs from experimentally changed compounds into precision-engineered medicines, harmonizing peptide structure with established antibacterial, safety, and translational performance profiles. These computational approaches signify a transition from post-hoc peptide optimization to prospective, mechanism-informed AMP design.

### Natural and Functional Immunological Role of Marine AMPs

Marine environments harbour some of the most diverse biological systems, and the organisms residing in these ecosystems—from unicellular microbes to complex vertebrates—have developed a diverse array of AMPs that are crucial components of their innate immune defense [344–347]. In marine microorganisms, such as bacteria, actinomycetes, and fungi, AMPs predominantly function as ecological regulators rather than traditional immune mediators. These peptides confer competitive benefits by suppressing adjacent microorganisms, regulating biofilm formation, and influencing local microbial communities [348, 349]. Numerous marine actinomycetes, for instance, produce formidable lipopeptides that enable them to prevail in nutrient-scarce environments, while also providing defense against bacteriophages and protozoan predators [350]. Upon isolation and application to mammalian systems, these peptides maintain significant functional efficacy, exhibiting membrane-disruptive antimicrobial characteristics, intracellular inhibition of microbial activities, notable anti-biofilm efficacy, and the ability to modulate immune responses by augmenting phagocyte activity and regulating cytokine release [351].

In marine crustaceans, AMPs assume a pivotal role due to the absence of adaptive immunity, relying predominantly on innate defenses. Crustacean AMPs, including penaeidins, crustins, and ALFs, are predominantly synthesised by haemocytes and swiftly released in response to microbial threats [66, 200, 352]. These peptides demonstrate extensive efficacy against bacteria, fungi, viruses, and parasites, while also modulating critical signalling pathways, including the Toll and immunological deficiency (IMD) pathways, which orchestrate antimicrobial gene expression [353, 354]. Among these molecules, ALFs are distinguished by their robust ability to bind and neutralize LPS, therefore averting dysregulated inflammatory or melanization reactions that could potentially harm host tissues [355]. In addition to their functions in direct microbial eradication, crustacean AMPs facilitate wound healing and regulate the prophenoloxidase system, hence assisting in the preservation of immunological homeostasis during infection and environmental stress. In mammalian models, many crustacean AMPs have immunomodulatory and anti-endotoxin properties, positioning them as potential options for anti-sepsis treatment, antiviral strategies, and vaccine adjuvant formulation [22, 356].

Marine algae generate peptide-based defense compounds that operate similarly to AMPs. Algal tissues, residing in environments perpetually subjected to microbial colonization, depend on these peptides to inhibit biofouling, mitigate opportunistic infections, and modulate oxidative stress responses. These peptides frequently fulfil several protective functions, acting as antimicrobial agents and as modulators of intracellular signalling related to stress tolerance and processes akin to programmed cell death. In mammalian systems, peptides derived from algae demonstrate significant antiviral, antibacterial, and antifungal properties, while also modulating immunological pathways such as nuclear factor kappa-light-chain-enhancer of activated B cells (NF-κB) and mitogen-activated protein kinase (MAPK) to elicit anti-inflammatory effects [357, 358]. Additionally, many algal peptides augment natural killer cell cytotoxicity and complement activation, hence enhancing immune surveillance, particularly against tumor cells [359, 360]. Their developing anticancer capabilities are ascribed to both direct production of apoptosis in malignant cells and the enhancement of host immune responses.

Fish among marine vertebrates generate some of the most functionally diverse and structurally robust AMPs known in aquatic ecosystems. Teleost AMPs—specifically piscidins, hepcidins, pleurocidins, and gaduscidins—are predominantly expressed in mucosal surfaces, including skin, gills, and the gastrointestinal tract, serving as an essential initial defense against pathogenic microorganisms [63, 361–363]. These peptides not only demonstrate direct antibacterial properties but also regulate the inflammatory response by modulating cytokine production, affecting neutrophil recruitment, and interacting with components of the adaptive immune system. Piscidins have remarkable structural stability across diverse salt and pH environments and have demonstrated the potential to augment antigen presentation and modulate B cell activation in mucosal tissues [65]. In mammalian systems, fish-derived AMPs maintain broad-spectrum antimicrobial efficacy and further stimulate immune-related processes [146, 364–369], such as macrophage respiratory burst activity, dendritic cell activation, wound healing, and angiogenesis [370]. Certain members of this category demonstrate specific cytotoxicity against altered cells, highlighting their potential as anticancer and immunotherapeutic drugs.

Marine AMPs, while mostly innate effector chemicals, have shown considerable ability to modulate T-cell-mediated immunity in mammalian systems. Numerous teleost-derived AMPs, including piscidins and pleurocidins, can directly influence lymphocyte function, augmenting T-cell activation or modifying cytokine release profiles pertinent to adaptive immunity [69, 270, 356, 370–373]. Certain pleurocidin analogues demonstrate preferential cytotoxicity towards tumor cells, promoting downstream T-cell recruitment and anticancer responses [374]. Marine AMPs significantly influence T cells indirectly by modulating antigen-presenting cells: piscidins promote dendritic cell maturation and enhance antigen-presenting capabilities [375, 376], whereas crustacean ALFs regulate cytokine production and neutralize endotoxins, thereby preserving T-cell activation during inflammatory stress [22, 355, 377]. Marine AMPs induce cytokines such as IL-12, IFN-γ, IL-6, and TGF-β, hence influencing Th1, Th2, or Th17 polarization and exhibiting more extensive immunomodulatory functions than previously acknowledged. In vaccine applications, certain marine AMPs serve as natural adjuvants that augment T-cell–dependent immunity, hence enhancing antigen-specific cellular responses in both fish and mammalian models [352]. Current evidence indicates that marine AMPs extend their biological functions beyond antimicrobial defense, serving as powerful modulators of both innate and adaptive immunity, with effects that directly or indirectly influence T-cell responses [63, 136].

**Fig. 4:**
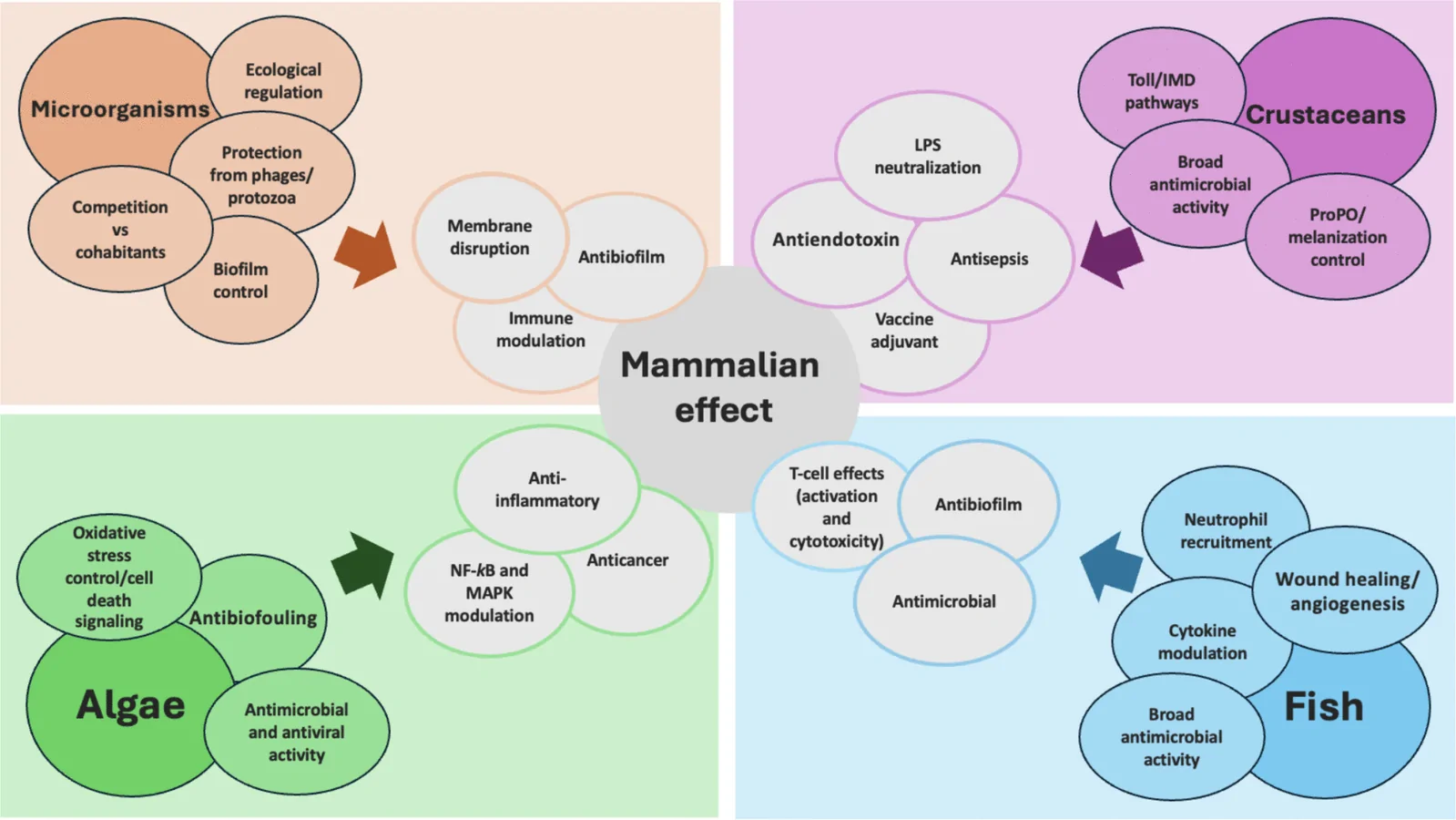
The immunological functions of marine antimicrobial peptides (AMPs) in their indigenous hosts and their therapeutic applications in mammalian systems. The upper right panel illustrates that in marine hosts, crustacean AMPs disrupt microbial membranes, bind to lipopolysaccharide (LPS), activate Toll and immune deficiency (IMD) signalling pathways, enhance phagocytosis and prophenoloxidase (proPO) activation, and assist in regulating melanization, thereby preserving immune homeostasis during infection. The lower right panel illustrates that fish mucosal surfaces generate AMPs that bolster barrier defense, augment cytokine release, and facilitate neutrophil recruitment when utilized in mammalian models, exhibit extensive antimicrobial and anti-biofilm efficacy while also influencing host immunity by augmenting phagocytosis, facilitating dendritic cell maturation and antigen presentation, and directing T cell responses and T helper (Th1/Th2/Th17) polarization via cytokines such as IL-12 and IFN-γ. In contrast, the lower left panel focuses on algal AMPs that inhibit biofouling and restrict colonization by opportunistic bacteria. The upper left panel indicates that marine microorganisms’ AMPs, which have anti-inflammatory and anticancer applications, also have indigenous antibiofouling and antimicrobial roles. Some AMPs bind to and neutralize endotoxins, protecting sepsis models. In contrast, others function as vaccine adjuvants that enhance antibody production and cellular responses, underscoring their promise as advanced anti-infective and immunotherapeutic agents

Marine AMPs collectively represent a highly diversified and evolutionarily refined molecular arsenal, influenced by the distinct selective pressures of aquatic habitats. Their remarkable structural flexibility, inherent stability under harsh environmental conditions, and dual aptitude for antibacterial action and immunological regulation underscore their significant therapeutic promise. In mammalian applications, these peptides function as potent antimicrobial agents and modulators of host immunity, altering cytokine networks, enhancing innate immune cell functions, inhibiting pathogenic biofilms, regulating inflammatory pathways, and amplifying vaccine-induced responses Fig. 4. Consequently, marine-derived AMPs present a viable source of next-generation immunotherapeutics for addressing infectious diseases, chronic inflammatory conditions, cancer, and antibiotic resistance.

## Applications of Marine AMPs in Biotechnology

### Biomedical Applications

Marine AMPs have intriguing prospects for the advancement of novel anti-infective medicines, given the rapid decline in efficacy of current antibiotics. Their ability to break microbial membranes through charge- and structure-specific interactions provides extensive efficacy against bacteria, fungi, viruses, and parasites, including MDR strains [54, 120, 378]. Numerous well-characterized marine peptides—including arenicins from *Arenicola marina* [133, 134], tachyplesins from horseshoe crabs [55, 379], myticins from mussels [143], and hedistin from polychaetes [38]—exhibit significant efficacy against MDR Gram-positive and Gram-negative bacteria.

In addition to their antibacterial properties, various marine AMPs demonstrate anticancer activity, such as kahalalide F derived from a mollusk-algal association [380, 381], halictin sourced from marine sponges, and epinecidin-1 obtained from groupers [120, 382], which selectively compromise tumor membranes or trigger apoptosis. Marine fish AMPs, including piscidin-1, piscidin-3, chrysophsin-1, and myxinidin, demonstrate potent antiparasitic activity against *Plasmodium* spp. and *Leishmania* spp. [223, 228, 255].

Numerous marine AMPs also influence host immunity; for instance, clavanins exhibit immunomodulatory and anti-endotoxin properties, mytilin B promotes immunological activation, and crustacean alpha-lactalbumin (α-LA) fragments neutralize endotoxin and mitigate systemic inflammation [184, 383]. Tachyplesins and invertebrate defensins exhibit significant endotoxin-neutralizing capabilities with therapeutic implications for sepsis [384]. These integrated activities underscore the biological potential of marine AMPs as versatile therapeutic agents.

### Aquaculture Applications

Aquaculture is a swiftly growing food production sector; yet it faces significant challenges from bacterial and fungal infections that threaten fish health and productivity [258]. Marine AMPs—including piscidins, epinecidin-1, moronecidin [224], chrysophsins [223], pleurocidin [232], hepcidins [385], and myxinidin [228]—exhibit potent activity against significant aquaculture pathogens such as *Vibrio*, *Edwardsiella*, *Aeromonas*, and *Streptococcus* spp. [382, 386]. Their ability to prevent biofilm development offers an additional advantage in hatchery settings, where persistent biofilms contribute to recurring disease outbreaks [387].

Crustacean AMPs, such as crustins [270], penaeidins [145], ALF [184, 388], and scygonadin [198], along with molluscan peptides like myticin C and mytilins, have demonstrated effectiveness as feed additives, immersion agents, or immune stimulants, rendering them essential assets in shrimp and shellfish AMP-based therapies diminish antibiotic consumption and impede the spread of AMR in aquaculture settings [280, 389]. The creation of AMP-enhanced meals, water treatments, and topical preparations demonstrates significant potential for sustainable disease management.

### Industrial Biotechnology Applications

Besides medicine and aquaculture, marine AMPs are increasingly acknowledged in industrial biotechnology. In the food industry, AMPs such as pleurocidin [232, 390], bacteriocin-like peptides from *Pseudoalteromonas* spp., and marine lipopeptides function as natural preservatives that inhibit foodborne pathogens and spoilage microorganisms while maintaining efficacy in high-salt environments commonly found in seafood preservation [391–393].

In industrial bioprocessing, AMPs offer eco-friendly alternatives for biofilm management. Tachyplesins exhibit significant anti-biofilm efficacy when immobilized on substrates [394], whilst arenicins have been included into anti-fouling coatings to diminish microbial adhesion [165]. Myxinidin and its derivatives have been effectively utilized in antimicrobial surface coatings for apparatus and bioreactors [226]. The incorporation of marine AMPs into filters, membranes, medical devices, and water treatment systems may significantly mitigate contamination concerns and enhance bioprocess reliability [395, 396].

### Integrative Outlook

The various applications collectively illustrate that marine AMPs extend beyond their fundamental protective roles to function as versatile biotechnological tools. The varied examples presented—such as piscidins, epinecidin-1, clavanins, tachyplesins, arenicins, pleurocidin, and crustins—demonstrate the extensive functional adaptability of marine peptides in biomedical, aquaculture, and industrial sectors. Their integration into clinical medicines, sustainable aquaculture, and antifouling technologies establishes them as pivotal elements of next-generation antimicrobial tactics [397–399]. With the rapid progression of peptide engineering, nanodelivery, and AI-assisted design, marine AMPs are set to play a crucial role in global health, food security, and industrial bioinnovation.

## AMP Resistance and Strategies to Overcome it

Although it has been assumed that AMPs won't yield widespread bacterial resistance because they tend to tackle the cell membrane, the discovery of specific protein targets, as will be discussed, raises the prospect of bacterial resistance and genetic changes. Some invasive bacteria, as well as those superficially residing within the host microbiome population, can cohabit with or even outcompete the host AMPs. Anti-therapeutic AMP defense mechanisms in the related bacteria may also be effective [297, 400, 401].

### AMP Resistance Mechanisms

#### Bacterial Sensing Systems

Bacteria can sense the presence of AMPs through sensors that trigger AMP-resistance mechanisms. The configuration and functionality of these sensors vary significantly between Gram-positive and Gram-negative bacteria [402]. Various AMP-resistance mechanisms encompass gene regulation, which must be executed autonomously due to its energy requirements and should only be activated in the presence of AMPs. AMP sensing may operate autonomously [403] or collaborate with additional bacterial resistance mechanisms. Significantly, some marine diseases have demonstrated the ability to detect and react particularly to marine AMPs. *Vibrio anguillarum*, for instance, activates PhoP/PhoQ-like systems and undergoes lipid A remodeling in response to teleost AMPs, such as piscidin-1 and epinecidin-1, thereby augmenting resistance to cationic peptides [382, 386]. Likewise, *Vibrio splendidus* and *Vibrio harveyi* identify bivalve AMPs, such as myticin C, and crustacean AMPs, including ALF, which activate two-component regulatory mechanisms that modify membrane charge and inhibit AMP binding [183, 303].

Similar to terrestrial bacteria, these systems relate to traditional sensor-regulator pathways. In Gram-negative bacteria, the AMP response is primarily mediated by two-component systems, [297, 403–405], while Gram-positive bacteria employ one-component, two-component, and occasionally multi-component signalling systems that work in concert to regulate AMP resistance pathways [297, 403, 406–413].

#### Protease-Based AMPs Resistance

Bacteria can inactivate AMPs by producing peptidases and proteases that cleave them at certain amino acid residues. Gram-positive bacteria neutralize AMP activity by secreting extracellular AMP-degrading proteases, but in Gram-negative bacteria, many proteases are situated in the OM or periplasmic space. Various bacterial proteases can effectively destroy AMPs by proteolytic digestion [414]. This mechanism is thoroughly established in marine systems,for instance, *Vibrio anguillarum* secretes metalloproteases such as EmpA that degrade fish AMPs, including piscidin-1 and piscidin-3, hence diminishing their bactericidal efficacy on mucosal surfaces [64, 415]. Similarly, *Vibrio splendidus* produces elastase-like proteases that can degrade mussel AMPs, such as myticin C, enabling the pathogen to circumvent hemocyte-mediated destruction [303, 416]. *Vibrio harveyi* in crustaceans secretes extracellular proteases that degrade shrimp AMPs, including ALF and penaeidins, hence compromising initial innate defenses [352, 417].

To mitigate proteolytic inactivation, enhanced resistance to protease degradation has been accomplished by replacing some or all L-amino acids in AMPs with D-amino acids [404, 408, 409, 418–424].

#### Remodelling and Modification of the Cell Membrane and the Cell Wall

The bacterial cell wall and membrane are primary targets of AMPs' bactericidal effect, wherein electrostatic interactions and pore creation result in swift cell death. Nevertheless, specific bacteria have developed resistance mechanisms by modifying cell surface elements, such as membrane charge, lipid composition, or surface polymers, which diminish the binding and effectiveness of AMPs [407, 409, 425].

##### Surface Charges Alteration

Bacteria can circumvent cationic AMPs by altering their surface charge to diminish electrostatic attraction. D-alanylation of teichoic acids (TA) and lipoteichoic acids (LTA) in Gram-positive bacteria reduces net negative charge, protecting cationic peptides [426, 427]. Gram-negative bacteria utilize similar mechanisms by modifying the phosphorylation, acylation, or sugar content of lipid A in LPS, consequently reducing AMP binding [428, 429]. Marine pathogens depend significantly on these modifications: *Vibrio anguillarum* modifies lipid A to withstand piscidin-1 exposure [63, 64], whereas *Vibrio harveyi* and *Vibrio splendidus* adjust LPS charge to avoid AMPs like epinecidin-1 and myticin C [303, 386]. These structural modifications constitute a significant impediment to AMP-induced membrane rupture.

##### Neutralization Process

A neutralizing process has been identified for the membrane lipids of many Gram-positive and Gram-negative bacteria, which mirrors the charge alteration of anionic teichoic acids mediated by D-alanylation. The cationic amino acid lysine is enzymatically conjugated to phosphatidylglycerol (PG), the predominant anionic phospholipid in bacterial membranes, through the multiple peptide resistance factor (MprF) system. The Lys-PG alteration alters the membrane surface charge from negative to positive, resulting in the electrostatic repulsion of cationic AMPs and significantly diminishing peptide binding [403, 408, 409, 412, 418].

Numerous marine AMPs are significantly influenced by this lysine-based charge reversal resistance mechanism. The teleost fish AMP piscidin-1, a highly cationic amphipathic α-helix, demonstrates decreased binding affinity to Lys-PG-enriched membranes, a characteristic associated with reduced pore formation [430]. Likewise, the β-sheet marine AMP tachyplesin I, sourced from the horseshoe crab *Tachypleus tridentatus*, exhibits markedly reduced membrane insertion upon interaction with MprF-mediated Lys-PG alteration in Gram-negative pathogens [379]. Tunicate-derived AMPs, such as clavanin A, have diminished efficacy against bacterial strains with elevated Lys-PG concentration, as the modified membrane charge obstructs their pH-dependent insertion [306]. These examples highlight that marine AMPs, despite their strong ability to target membranes, are vulnerable to bacterial lipid remodelling tactics that alter membrane electrostatics.

##### Alterations in Membrane Lipid Composition

A notable resistance mechanism emerges when AMPs penetrate extracellular barriers and access the cytoplasmic membrane, which is the principal locus of antimicrobial action. Due to the presence of anionic head groups in most bacterial membrane phospholipids, they typically attract cationic AMPs, hence promoting electrostatic binding and membrane insertion. Bacteria often alter their membrane lipid composition to diminish AMP affinity. A notable instance, especially in *Staphylococcus aureus*, is the lysylation of PGN through the MprF system, which transforms PGN into cationic Lys-PG, consequently diminishing the membrane's net negative charge and impeding the binding and incorporation of cationic AMPs [406, 431].

In Gram-negative bacteria, resistance is augmented through lipid A remodelling, which encompasses acylation, palmitoylation, or the addition of phosphoethanolamine, all of which boost the hydrophobicity and density of the OM, hence reducing AMP penetration [432, 433]. These phospholipid modifications exemplify a conserved bacterial mechanism to counteract membrane-targeting AMPs. Several marine AMPs, including the strongly cationic teleost peptides piscidin-1 and piscidin-3, along with β-sheet AMPs such as tachyplesin I from the horseshoe crab, demonstrate significantly diminished binding and pore-forming abilities in bacteria with elevated levels of Lys-PG or acylated lipid A. This illustrates how lipid remodelling directly reduces the efficacy of otherwise potent marine AMPs [224, 379, 430].

##### Changes in Membrane Fluidity

A distinct membrane-based mechanism of AMP resistance entails the alteration of OM lipid composition, specifically by lipid A acylation in Gram-negative bacteria. These modifications not only reduce membrane permeability but also modify the biophysical characteristics of the OM. The integration of long-chain fatty acids or supplementary secondary acyl chains into lipid A in numerous pathogens diminishes membrane permeability, leading to denser lipid packing that hinders AMP insertion [409, 433]. Certain bacteria also integrate unsaturated or branched-chain fatty acids, facilitating the regulated adjustment of membrane viscosity to mitigate AMP-induced disarray [434]. These alterations jointly reinforce the OM and impede the interaction of cationic AMPs with lipid A.

This resistance mechanism has been shown to diminish the effectiveness of many marine AMPs, including the α-helical fish peptides piscidin-1 and piscidin-3, whose deep membrane insertion is notably hindered when OM fluidity is decreased due to lipid A acylation [430]. Likewise, the β-sheet peptide tachyplesin I from horseshoe crabs, which typically destabilizes lipid domains, exhibits reduced binding affinity to altered, rigidified LPS structures [379]. These examples demonstrate that bacterial modulation of membrane fluidity directly diminishes the permeability and antibacterial efficacy of marine-derived AMPs.

#### Active Efflux Pump-Mediated AMPs Resistance

Similar to traditional antibiotics, intracellularly acting AMPs are susceptible to active efflux mechanisms that transport peptides from the periplasm or cytoplasm prior to their interaction with intracellular targets [297, 401, 435]. Marine-derived AMPs that depend on intracellular lethality, such as pleurocidin, which infiltrates bacterial cells and interferes with nucleic acid-related functions, have less efficacy in strains that overexpress resistance nodulation cell division (RND)-subfamily efflux pumps [231, 232]. Likewise, the marine peptide myxinidin, recognized for its ability to permeabilize membranes and subsequently alter intracellular structures, demonstrates diminished effectiveness against efflux-competent Gram-negative bacteria [226, 228].

To mitigate efflux-based resistance, one approach entails the engineering of efflux-resistant surface-active AMPs. Screening methods utilizing immobilized AMPs have demonstrated efficacy in discovering peptides that maintain their action despite depletion caused by efflux mechanisms [436–438]. The advancement of nanoparticle- or microparticle-anchored AMPs presents a promising opportunity: when AMPs are affixed to solid surfaces, they evade intracellular accumulation and consequently circumvent efflux mechanisms entirely [407, 418, 439, 440]. These techniques may be particularly advantageous for marine AMPs with strong membrane-disruptive properties—such as piscidin-1 and tachyplesin I—since immobilization could maintain their bactericidal efficacy while inhibiting efflux-mediated resistance [224, 379].

#### Entrapment by Surface Proteins and Polysaccharides

Some bacterial species evade AMP attacks by producing surface-anchored or secreted proteins and polysaccharides that demonstrate a strong binding affinity for these peptides. These decoy molecules act as molecular "sinks," sequestering AMPs prior to their arrival at the cytoplasmic membrane. The capsular polysaccharides of *Klebsiella pneumoniae* and *Streptococcus pneumoniae* can bind to and neutralize host defensins, hence reducing peptide accessibility to the bacterial surface [441–443]. Comparable binding effects have been recorded for staphylokinase, which sequesters human α-defensins to shield *Staphylococcus aureus* from AMP-mediated lethality [425].

Marine AMPs, particularly highly cationic β-sheet peptides, are susceptible to sequestration by bacterial extracellular polymers. The marine peptide tachyplesin I, sourced from the horseshoe crab *Tachypleus tridentatus*, exhibits a considerable affinity for LPS-rich extracellular components of Gram-negative pathogens, hence restricting its effective concentration at the membrane [146, 379]. Likewise, piscidin-1, a powerful teleost AMP, can be sequestered by bacterial extracellular polysaccharides and biofilm matrix, diminishing its efficacy against encapsulated pathogens [64, 132]. These examples demonstrate that binding-based evasion—through capsules, LPS, or secreted proteins—represents a significant bacterial approach to neutralize both mammalian and marine-derived AMPs.

##### Biofilm Formation

Bacteria in biofilms are encased in a self-generated extracellular matrix predominantly consisting of polysaccharides, extracellular DNA (eDNA), and proteins, which obstructs AMPs from accessing the bacterial cell surface. This matrix can electrostatically confine cationic AMPs, impede their diffusion, or repel them via dense negative charges, consequently diminishing peptide accessibility to embedded cells. Thus, merely a minority of identified AMPs demonstrate significant antibiofilm efficacy [123, 127, 422, 436–438, 444, 445].

Numerous marine AMPs exhibit remarkable antibiofilm capabilities that enable them to surpass matrix-mediated sequestration. The peptide octominin, derived from the cephalopod *Octopus minor*, compromises biofilm structure and diminishes biomass in *Acinetobacter baumannii* and *Candida albicans* via membrane permeabilization and extracellular DNA degradation [121, 122, 124, 125]. Peptides from teleost fish, including piscidin-1 and epinecidin-1, exhibit significant antibiofilm activity against Gram-negative and Gram-positive pathogens by infiltrating biofilm matrices and inducing oxidative stress [132, 386]. Similarly, the arenicins derived from the annelid *Arenicola marina* can destabilize polysaccharide-rich matrices and diminish biofilm viability [165].

Due to the challenges associated with eliminating mature biofilms, recent studies have focused on the creation and engineering of AMPs specifically designed to inhibit biofilm formation or disrupt established biofilms [408, 409, 418, 446, 447]. Marine AMPs, due to their structural diversity, elevated cationic charge, and salt-stable conformations, serve as promising frameworks for advanced antibiofilm therapies.

##### Capsular Exopolymers Formation

A further mechanism of bacterial resistance to AMPs entails the synthesis of long-chain capsular polysaccharides and exopolymers that encase the OM, serving as physical and electrical barriers. These capsules can create thick, highly anionic matrices that bind and sequester cationic AMPs prior to their interaction with the bacterial cell surface. While not all capsule-producing species demonstrate significant AMP resistance, the level of protection typically correlates with capsule thickness and the concentration of negatively charged residues within the exopolysaccharide matrix [407, 418, 429, 434, 441, 443, 448, 449].

Marine AMPs exemplify how certain peptides can bypass or evade capsule-mediated sequestration. The cationic β-sheet peptide tachyplesin I, derived from *Tachypleus tridentatus*, exhibits great binding affinity for LPS and may permeate polysaccharide-rich surfaces, hence maintaining its efficacy against encapsulated Gram-negative bacteria [55, 379]. The teleost peptide piscidin-1, characterized by significant membrane affinity and metal-chelating capabilities, has shown efficacy against encapsulated bacteria like *Streptococcus iniae*, suggesting its capacity to engage with and penetrate protective polysaccharide layers [132]. Arenicins from *Arenicola marina* exhibit salt-stable, high-affinity binding to anionic extracellular components, facilitating the disruption of biofilms and capsule-like extracellular matrices [165]. These examples demonstrate the ability of certain marine AMPs to maintain efficacy despite the presence of dense capsular exopolymers that usually obstruct conventional AMPs.

#### Cross-Resistance with Host-Defense Peptides

The possibility of cross-resistance between therapeutic AMPs and endogenous host-defense peptides (HDPs) has elicited apprehension about the potential for exposure to one class of peptides to reduce bacterial susceptibility to another. This concept posits that bacteria subjected to therapeutic AMPs—either synthetic or naturally sourced—may develop adaptations that diminish their susceptibility to human innate immune peptides, including LL-37 or defensins [450, 451]. Comparable apprehensions pertain to the utilization of marine-derived AMPs as medicinal agents. Bacterial adaptation to the highly cationic marine peptides piscidin-1 and tachyplesin I, which possess structural similarities to mammalian α-helical or β-sheet AMPs, may theoretically affect susceptibility to human host defense peptides [55, 224]. Similarly, resistance mechanisms activated against marine AMPs, such as epinecidin-1—such as alterations in membrane charge or efflux—mechanistically coincide with those employed to resist LL-37 [382], indicating potential but unverified cross-resistance associations.

Notwithstanding these apprehensions, empirical evidence demonstrates that AMP resistance develops at low frequencies and does not universally provide extensive cross-resistance to host peptides. Dobson et al. [450] demonstrated that AMP-resistant mutants emerge infrequently compared to antibiotic-resistant mutants and that resistant variants frequently incur fitness costs that restrict their survival. Likewise, current research fails to show a consistent disparity between AMPs and traditional antibiotics regarding the long-term survival or pathogenicity of resistant bacteria in vivo [452]. Collectively, these findings indicate that although theoretical mechanisms for cross-resistance are present, the clinical risk is minimal, and the therapeutic application of AMPs—particularly those derived from marine sources—does not presently seem to undermine host innate immunity.

#### Modulation of Host AMP Gene Expression

The ultimate mechanism of bacterial resistance to AMPs entails modifying the expression of the host's innate immune genes. Numerous human diseases diminish the expression of LL-37 and β-defensin-1 to lower mucosal AMP levels [453, 454]. Comparable tactics are seen in marine ecosystems: pathogenic *Vibrio* spp. inhibit the transcription of essential teleost AMPs, including piscidin-1 and piscidin-3 [382, 386], epinecidin-1 [120], and hepcidins [307, 385]. In invertebrates, diseases such as *Vibrio splendidus* suppress myticin C in mussels [303], whereas shrimp pathogens impede ALF gene expression to compromise initial immune responses [183, 184]. These interactions illustrate that microbial pathogens from many marine species can influence host AMP gene expression to evade innate immune responses.

### Strategies to Overcome AMP Resistance

The effort to overcome the challenges of utilizing AMPs as therapeutic alternatives is warranted due to their broad-spectrum efficacy, swift bactericidal properties, and reduced likelihood of resistance development compared to traditional antibiotics [455, 456]. Nonetheless, pathogens have developed many responses, such as membrane remodelling, surface charge alteration, proteolytic degradation, and AMP sequestration, which can reduce peptide effectiveness [407, 409]. The clarification of these resistance routes has expedited the formulation of logical engineering solutions to enhance AMP stability, selectivity, and therapeutic efficacy. C-terminal amidation, N-terminal acetylation, D-amino-acid substitution, and deliberate modifications in charge and hydrophobicity can significantly improve protease resistance, diminish cytotoxicity, and augment microbial selectivity [457, 458].

In addition to structural optimization, the modification of biochemical characteristics—such as cationicity, hydrophobicity, amphipathicity, and helix propensity—continues to be a fundamental design principle. Moreover, AMPs may demonstrate synergistic effects with traditional antibiotics, offering a means to reduce necessary drug dosages and inhibit the emergence of resistance [338, 459, 460]. Synergy has been empirically established for AMPs in conjunction with β-lactams, fluoroquinolones, aminoglycosides, and sulfonamides. Ampicillin-induced decrease of surface charge increases sensitivity to daptomycin and other cationic peptides [459]. The amalgamation of synthetic cationic peptides with penicillin, ceftazidime, norfloxacin, or ciprofloxacin has demonstrated significant synergy against multidrug-resistant *Staphylococcus aureus*, *Pseudomonas aeruginosa*, *Klebsiella pneumoniae*, and *Escherichia coli* [459]. Similarly, streptomycin and cotrimoxazole exhibit a synergistic effect with cationic peptides against resistant clinical isolates of *Brucella melitensis* [461]. These findings collectively underscore that peptide engineering and combinatorial techniques, particularly those guided by naturally robust marine AMP scaffolds, substantially enhance the translational potential of AMPs, as illustrated in Table 3.

**Table 3:** AMPs’ resistance mechanisms and biotechnological design responses

Resistance mechanism	Design workaround	References
Protease degradation	Incorporation of D-amino acids, peptide cyclization, terminal amidation/acetylation	[217, 276]
Surface charge modification	Sequence engineering to optimize charge balance; incorporation of non-natural residues	[462, 463]
Outer membrane barrier	Use of permeabilizers, nanocarriers (liposomes, polymeric nanoparticles) to enhance uptake	[217, 464]
Biofilm shielding	Liposomes/hydrogels for penetration + quorum-sensing interference strategies	[419, 465]
Efflux pumps	Multidrug efflux pump inhibitors; design of shorter, more hydrophobic analogs	[466]
Host toxicity / hemolysis	Structure–activity redesign (hydrophobicity tuning, truncated analogs) to enhance selectivity	[462, 463]

#### Amino Acid Modifications for Protease Resistance

A primary restriction of natural AMPs is their swift breakdown by host and microbial proteases, significantly reducing their therapeutic half-life and constraining systemic application. A diverse array of amino acid changes and chemical modifications has been employed to improve protease resistance and mitigate instability. This encompasses the integration of D-amino acids, cyclization of the peptide backbone, sequence truncation, and terminal alterations like N-acetylation or C-terminal amidation. These techniques can stabilize the peptide's secondary structure, diminish recognition by proteases, and preserve or potentially augment antibacterial action.

Marine AMPs exemplify how structural engineering can significantly enhance stability. Tachyplesin I, a β-sheet peptide derived from *Tachypleus tridentatus*, has been effectively changed using D-amino-acid substitution and disulphide engineering to produce analogues exhibiting enhanced serum stability and preserved antibacterial activity [55, 467, 468]. Piscidin-1 and epinecidin-1, two highly cationic AMPs produced from teleosts, have demonstrated notable enhancements in protease resistance and decreased cytotoxicity through terminal amidation or deliberate residue substitution [382, 469]. Likewise, structure-guided engineering of epinecidin-1 from *Epinephelus coioides* has produced variants exhibiting improved protease resistance and anti-MDR action [382]. β-strand marine peptides, such as arenicin-1 derived from *Arenicola marina*, have been modified through sequence truncation and structural rigidification to reduce proteolytic cleavage while maintaining potent membrane-disruptive action [165].

These engineering methodologies—exemplified in both classical and marine AMPs—highlight the potential to convert naturally protease-sensitive molecules into clinically applicable therapeutic agents [455, 456, 458, 470, 471].

#### Conjugation with Nanoparticles for Delivery

Nanotechnology provides intriguing methods for improving the stability, selectivity, and delivery of AMPs. Conjugation with nanomaterials, such as liposomes, polymeric nanoparticles, metallic nanoparticles, nanogels, and stimuli-responsive hydrogels, can protect peptides from enzymatic degradation, prolong circulation time, and facilitate controlled release at infection sites. Nanocarriers enable precise distribution to bacterial cells or biofilms, minimizing off-target cytotoxicity and enhancing therapeutic efficacy. AMP-loaded liposomes penetrate biofilms more effectively than free peptides, delivering 5–tenfold greater local peptide concentrations at infection sites while reducing systemic toxicity in vivo [472–474].

Marine-derived AMPs are formidable prospects for nanodelivery owing to their significant efficacy and salt-stable configurations. Liposomal encapsulation of piscidin-1 augments membrane insertion and diminishes haemolytic toxicity [475], whereas polymeric nanoparticle formulations of epinecidin-1 boost peptide stability and extend antibacterial efficacy against Gram-negative infections [382]. Metallic nanoparticles linked with β-sheet marine peptides like arenicin-1 have synergistic antibacterial and antibiofilm properties, resulting from the dual mechanisms of membrane rupture and nano-facilitated reactive oxygen species (ROS) production [165]. Moreover, myxinidin, sourced from hagfish, exhibits markedly improved protease resistance and biofilm infiltration when administered by chitosan-based nanocarriers [226].

Collectively, these nanoscale approaches signify a significant biotechnological progression that enables the conversion of AMPs, including marine peptides, into clinically viable anti-infective agents [472, 476–481].

#### Synergistic AMP–Antibiotic Therapies

A further biotechnological approach to enhance the efficacy of AMPs is their integration with traditional antibiotics. AMPs can compromise bacterial membranes and diminish surface charge, hence enhancing pathogen susceptibility to antibiotic penetration and efficacy. This synergy boosts antimicrobial activity while decreasing the necessary dosages of both drugs, hence minimizing potential toxicity and delaying the development of resistance. Numerous marine-derived AMPs have exhibited strong antibiotic-synergistic properties. Piscidin-1 markedly augments the efficacy of oxytetracycline and florfenicol against multidrug-resistant *Vibrio* spp. by enhancing membrane permeability [65, 482]. Epinecidin-1 derived from grouper demonstrates synergistic antibacterial properties when used in conjunction with aminoglycosides and β-lactams, due to AMP-facilitated augmentation of antibiotic infiltration into Gram-negative bacteria [382]. Arenicin-1, a β-sheet peptide derived from lugworm, exhibits synergistic effects with ciprofloxacin and rifampicin against *Pseudomonas aeruginosa* biofilms through collaborative membrane disruption and enhanced intracellular antibiotic penetration [165]. Experimental investigations on synthetic and non-marine AMPs corroborate these findings, demonstrating significant synergistic effects between cationic peptides and antibiotics such as penicillins, cephalosporins, and fluoroquinolones against multidrug-resistant pathogens, including *Staphylococcus aureus*, *Pseudomonas aeruginosa*, and *Klebsiella pneumoniae* [457, 483–488].

Amino acid changes, nanocarrier-based delivery systems, and synergistic AMP-antibiotic therapy collectively demonstrate how biotechnology may address the inherent limits of marine AMPs. These methods enhance peptide stability and bioavailability while broadening their therapeutic applications through increased potency and diminished resistance development. The integration of peptide engineering with nanotechnology and combinatorial pharmacology establishes a translational framework for transforming marine AMPs from experimental substances into effective antibacterial drugs. As these strategies advance, they will be crucial in connecting marine biodiversity with clinically effective therapeutics [397, 399, 489–494].

## Translational and Industrial Barriers in Marine AMP Development

Despite the significant antibacterial potential of marine AMPs, their incorporation in clinical and industrial applications is hindered by non-molecular obstacles that surpass peptide design. Although resistance mitigation strategies such as sequence modification, nanodelivery, and combinatorial therapies are thoroughly examined, economic viability, scalable production, regulatory-compliant quality assurance, and developmental efficiency now serve as the primary factors influencing the success or failure of AMP during late-stage development [389, 398, 456, 490, 491, 493, 495–497].

### Economic Cost and Production Feasibility

A significant challenge in AMP translation is the production cost, which greatly affects economic feasibility. The direct extraction of marine species is fundamentally unsustainable and lacks reproducibility, whereas SPPS becomes too costly for longer, modified, or cysteine-rich peptides. SPPS is projected to be 10–20 times more expensive than fermentation-based recombinant synthesis on a per-gram basis, especially when pharmaceutical-grade purity and regulatory documentation are necessary [399, 498, 499]. These economic limitations particularly hinder systemic applications necessitating high or repeated dosing, thus promoting the early adoption of biosynthetic production platforms during candidate selection [398, 456, 500–505].

### Scalability and Chemistry, Manufacturing, and Controls (CMC)

In addition to economic factors, scalability and CMC compliance represent significant translational obstacles for marine AMPs. Recombinant expression in bacterial, yeast, or eukaryotic systems provides scalable options but presents obstacles such as host toxicity, peptide aggregation, proteolytic degradation, batch-to-batch variability, and intricate downstream purification [499, 506]. Furthermore, regulatory standards for good manufacturing practice (GMP) production necessitate stringent oversight of peptide identity, purity, structural integrity, stability, and degradation products—criteria that are especially rigorous for cationic and amphipathic peptides susceptible to aggregation and adsorption losses [310, 398, 399, 497, 507–509]. Neglecting to resolve these CMC problems early in development often leads to late-stage attrition, regardless of antibacterial efficacy.

### AI and ML as Translational Enablers

In recent years, AI and ML have emerged as revolutionary instruments that can decrease development costs, expedite optimization, and enhance the manufacturability of advanced manufacturing processes. ML algorithms are currently commonly utilized to forecast antimicrobial activity, toxicity, solubility, aggregation tendency, protease susceptibility, and PK behaviour directly from peptide sequence data [46, 47, 510]. AI-assisted design facilitates the prioritization of shorter, structurally simpler, and more stable peptide designs, resulting in reduced synthesis costs and enhanced scalability [511, 512]. In this context, AI serves not only as a catalyst for discovery but also as a strategic facilitator of cost-efficient, CMC-ready AMP development, aiding in the transition from laboratory innovation to industrial implementation.

### Perspective and Outlook

Marine AMPs should be regarded within a systems-level translational paradigm that integrates marine biodiversity with synthetic biology, scalable manufacturing, regulatory science, and computational optimization. Although immediate applications in aquaculture, food safety, and topical formulations may occur before systemic clinical use, wider therapeutic implementation will rely on the simultaneous resolution of economic limitations, CMC scalability, and PK/PD optimization, in conjunction with antimicrobial efficacy [251, 506, 513]. Ongoing multidisciplinary collaboration across marine biology, biotechnology, pharmacology, and AI will be crucial to convert marine AMP diversity into therapeutically and industrially applicable anti-infective therapies that can effectively combat AMR on a large scale.

## Conclusion

Marine AMPs are evolutionarily conserved molecules that form a vital first line of defense across diverse organisms. Their cationic and amphipathic properties enable selective disruption of microbial membranes, while their multifunctionality—including immunomodulatory, antiviral, anticancer, and antiparasitic activities—underscores their value as candidates for next-generation therapeutics and biotechnological applications. Despite rapid advances, key barriers remain: the low abundance of AMPs in marine organisms, challenges in large-scale isolation, and limitations such as hemolysis, proteolytic degradation, and poor PK. These constraints highlight the need for innovative biotechnological approaches. Recombinant expression, peptide engineering, nanodelivery systems, and omics-guided discovery are already enhancing stability, scalability, and functional optimization, extending AMP use beyond human medicine to aquaculture, food preservation, and industrial biofilm control. Looking forward, the integration of marine biotechnology with synthetic biology, nanotechnology, and computational peptide design provides a clear pathway for overcoming current challenges. By converting naturally occurring AMPs into engineered, stable, and cost-effective molecules, these approaches can establish marine AMPs as a cornerstone in the global fight against AMR. Achieving this vision will depend on sustained multidisciplinary collaboration that bridges marine biology, biotechnology, and translational medicine. Quantitative advances in yield, cost reduction, and PK highlight that marine AMPs are moving beyond exploratory biology toward feasible translational pipelines.

## Data Availability

No datasets were generated or analysed during the current study.
